# HURP localization in metaphase is the result of a multi-step process requiring its phosphorylation at Ser627 residue

**DOI:** 10.3389/fcell.2023.981425

**Published:** 2023-07-05

**Authors:** Stylianos Didaskalou, Christos Efstathiou, Sotirios Galtsidis, Ilοna Kesisova, Aliaksandr Halavatyi, Tountzai Elmali, Avgi Tsolou, Andreas Girod, Maria Koffa

**Affiliations:** ^1^ Department of Molecular Biology and Genetics, Democritus University of Thrace, Alexandroupolis, Greece; ^2^ Department of Life Sciences and Medicine, University of Luxembourg, Esch-sur-Alzette, Luxembourg; ^3^ Advanced Light Microscopy Facility, European Molecular Biology Laboratory, Heidelberg, Germany

**Keywords:** HURP dynamics, HURP localization, HURP phosphorylation, FRAP (fluorescence recovery after photobleaching), photoactivation, mitosis, Aurora A, TPX2

## Abstract

Faithful chromosome segregation during cell division requires accurate mitotic spindle formation. As mitosis occurs rapidly within the cell cycle, the proteins involved in mitotic spindle assembly undergo rapid changes, including their interactions with other proteins. The proper localization of the HURP protein on the kinetochore fibers, in close proximity to chromosomes, is crucial for ensuring accurate congression and segregation of chromosomes. In this study, we employ photoactivation and FRAP experiments to investigate the impact of alterations in microtubule flux and phosphorylation of HURP at the Ser627 residue on its dynamics. Furthermore, through immunoprecipitations assays, we demonstrate the interactions of HURP with various proteins, such as TPX2, Aurora A, Eg5, Dynein, Kif5B, and Importin *β*, in mammalian cells during mitosis. We also find that phosphorylation of HURP at Ser627 regulates its interaction with these partners during mitosis. Our findings suggest that HURP participates in at least two distinct complexes during metaphase to ensure its proper localization in close proximity to chromosomes, thereby promoting the bundling and stabilization of kinetochore fibers.

## Introduction

Proper mitotic spindle assembly and function is essential for faithful chromosome segregation into the two daughter cells during cell division. Even minor alterations of the mitotic spindle can lead to an increased rate of chromosome missegregation, a process described as Chromosomal Instability (CIN) (Gordon et al., 2012). The main consequence of CIN is aneuploidy, a state of a cell containing an abnormal number of chromosomes or parts thereof. Aneuploidy itself seems to lead to increased genomic instability (Potapova et al., 2013; Giam and Rancati, 2015). Defects in mitotic spindle assembly or dynamics, incorrect chromosome congression, defective mitotic checkpoint and improper kinetochore-microtubule attachment can all lead to aneuploidy and, in turn, to increased chromosomal instability.

The mitotic spindle responsible for the accurate segregation of chromosomes consists of microtubules (MTs), molecular motor proteins and Microtubule Associated Proteins (MAPs). Mitotic spindle formation is regulated by the small Ran-GTPase which forms a gradient surrounding chromosomes (Kalab et al., 2002), releasing spindle-assembly factors (SAFs) from the inhibitory binding of Importins α/β at the chromosome vicinity (Nachury et al., 2001; Wiese et al., 2001; Gruss et al., 2002), although a recent study showed that some SAFs localize on the mitotic spindle independently of the Ran-GTP (Tsuchiya et al., 2021). Several SAFs have been identified, such as TPX2, NuMA, NuSAP, Aurora A, HURP and CHD4 (Gruss et al., 2002; Kufer et al., 2002; Yu et al., 2005; Koffa et al., 2006; Santarella et al., 2007; Yokoyama et al., 2013).

HURP (Hepatoma Upregulated Protein) was identified as a protein overexpressed in hepatocellular carcinoma, representing a potential oncogene (Tsou et al., 2003). HURP is overexpressed in many other cancer types such as bladder, breast, glioblastoma, prostate and non-small cell lung cancer and has been proposed as a new cancer biomarker (Chen et al., 2014; Eissa et al., 2014; Stangeland et al., 2015; Espinoza et al., 2016; Tagal et al., 2017).

We and others have shown that HURP bundles and stabilizes kinetochore fibers (kt-fibers), and is essential for proper chromosome congression and segregation during cell division (Koffa et al., 2006; Sillje et al., 2006; Wong and Fang, 2006; Santarella et al., 2007). Our immunoprecipitation experiments in *Xenopus* egg extracts demonstrated that HURP interacts with other MAPs, such as TPX2, XMAP215, Eg5, and Aurora A (Koffa et al., 2006), forming a complex required for Ran-dependent bipolar spindle formation.

In mammalian cells, TPX2 directly interacts with, Eg5 (Eibes et al., 2018), and is required for the activation of the mitotic Aurora A kinase (Bayliss et al., 2003). HURP is phosphorylated by Aurora A, increasing its affinity for MTs as well as its stability (Yu et al., 2005). HURP preferentially localizes at chromosome-proximal regions of kt-fibers and this preference is regulated by Aurora A (Wong et al., 2008; Kesisova et al., 2013). However, both TPX2 and Aurora A show a different localization from HURP, with a preference towards the spindle poles (Gruss et al., 2002; Kufer et al., 2002).

The aforementioned findings suggest that, in order to achieve a proper mitotic spindle in mammalian cells, Ran-regulated SAFs may interact with each other in a very distinct pattern in time and space. Moreover, HURP may participate in more than one complexes, transiently formed during cell division, until it reaches its final chromosome-proximal destination.

Το explore these possibilities, we examined the spatiotemporal dynamics of HURP in metaphasic cells by Fluorescence Recovery After Photobleaching (FRAP) and Photoactivation (PA) experiments. FRAP experiments showed that HURP dynamics are affected upon MT flux alterations and its phosphorylation at the Ser627 residue, while PA experiments showed a bi-directional movement of HURP molecules on spindle MTs. Finally, immunoprecipitation experiments showed that HURP interacts with motor proteins that could explain the observed kinetics.

## Materials and methods

### Generation of eGFP-HURP HeLa Kyoto cell line

In order to generate the eGFP-HURP HeLa Kyoto cell clones we followed the protocol published by Koch et al. (2018). Briefly, we have used the paired Cas9D10A nickase approach to insert eGFP tag upstream of the N-terminus of the HURP endogenous gene via homology-directed repair. The sequencing data of the HURP gene, as well as its upstream and downstream sequences in HeLa Kyoto cells, was kindly provided by Professor Ellenberg and used in order to design the gRNAs and donor plasmid. The design of gRNA oligos was done using the CRISPR Guide RNA Design services of (benchling.com) and the synthesis of the gRNA oligos (Table 1) and donor plasmid was done using the services of Genecust (genecust.com). These gRNA oligos were cloned into the pX335-U6-Chimeric_BB-CBh-hSpCas9n (D10A) vector (Addgene plasmid ID#42335) using the FastDigest BbsI (BpiI) (Thermo Scientific, cat. No. FD1014). The donor plasmid (HURP donor plasmid) contains the template for homologous recombination with the fluorescent marker gene (eGFP) flanked by 800 bp homology arms complementary to the target site of HURP. Clones that expressed the fusion protein at the correct sub-cellular localization as determined by fluorescence microscopy were taken into culture and were analyzed by genomic PCR and Western blot for correct integration of the construct and expression of the fusion proteins. Only heterozygous clones expressing eGFP-HURP fusion proteins were found.

**TABLE 1:** Information of gRNA oligos used to generate the eGFP-HURP HeLa Kyoto cell line.

Name	Locus	Sequence	Plasmid
gRNA oligo sense	gRNA_HURP_46s	cac​cgA​AGA​CAT​CCT​GTC​AAG​GAA​A	gRNA_HURP_46s and gRNA_HURP_46a oligos
gRNA oligo antisense	gRNA_HURP_46a	aaa​cTT​TCC​TTG​ACA​GGA​TGT​CTT​c	annealed and cloned into the px335 plasmid
gRNA oligo sense	gRNA_HURP_94s	cac​cgC​ACA​TTT​TGC​CAG​TCG​ACA​C	gRNA_HURP_94s and gRNA_HURP_94a oligos
gRNA oligo antisense	gRNA_HURP_94a	aaa​cGT​GTC​GAC​TGG​CAA​AAT​GTG​c	annealed and cloned into the px335 plasmid

### Cell culture and synchronization conditions

HeLa Kyoto cells were maintained either in DMEM-high glucose (GIBCO) or in RPMI-1640 (Pan Biotech) supplemented with 10% FBS (GIBCO), 2 mM L-glutamine (Lonza), 100 U/mL penicillin and 100 μg/mL streptomycin (Invitrogen) at 37°C with 5% CO_2_ in a humidified incubator. For metaphase synchronization, cells treated with 50 ng/mL Nocodazole (Sigma) for 6 h, after thorough washing with PBS (1-3 times), were released in growth medium containing 10 μM MG132 (Calbiochem) and assayed 2 h later. No effect of MG132 treatment on MT dynamics was found, assessed by measuring inter-kinetochore distances in synchronous and asynchronous metaphasic HeLa Kyoto cells (Supplementary Figure S1A).

### HURP WT construct and site directed mutagenesis

HURP CDS corresponding to RefSeq NM_014750.4 was obtained from the IMAGE clone (IRATp970H058D). HURP WT (1-846aa) construct was created by cloning into pEGFP-C3 plasmid using HindIII/BamHI restriction sites. Primer sequences were as follows: forward 5′-AAA​AAA​AAG​CTT​ATG​TCT​TCA​TCA​CAT-3′ and reverse 5′- TTT​TTT​GGA​TCC​TCA​AAA​TTC​TCC​TGG​TTG​TAG-3’. Site directed mutagenesis was performed using KAPA HiFi™ DNA Polymerase (KAPABIOSYSTEMS) according to the manufacturer’s instructions. The pEGFP-C3 plasmid was used as a template to introduce mutations in the coding sequence of HURP and alter the Serine residues 627, 725, 757 and 830 to Alanine (S627A, S725A, S757A, S830A and 4P) using the primers 5′-GTT​AAA​TTA​TTC​TCA​GGA​CTT​GCT​GTC​TCT​TCT​GAA​GGC​C-3′, 5′-GTT​TAT​CCA​GTG​AGA​GAA​TGG​CTT​T GCC​TCT​TCT​TGC​TGG​TG-3′, 5′-AAA​CAT​CCT​GTG​ATG​TAA​TTG​CAG​AAT​TCA​GTT​CCA​TTC​CTT​CC-3′, 5′-GAACATG CCA​GAC​ACA​TTG​CTT​TTG​GTG​GTA​ACC​TGA-3′, respectively. Serine residue 627 was also altered to Aspartic acid (S627D) using the following primer 5′-GTT​AAA​TTA​TTC​TCA​GGA​CTT​GAT​GTC​TCT​TCT​GAA​GGC​C-3′. PCR mutagenesis was set up according to the supplier’s instructions and annealing was performed at 70°C. Digestion with *Dpn*l was carried out using 10U of enzyme per reaction (New England Biolabs), followed by ligation reaction with KAPA Rapid Ligation System (KAPABIOSYSTEMS), following the supplier’s instructions. All clones were verified by sequencing.

### Transfection conditions

For immunofluorescence, cells were transfected with different HURP constructs: WT, S627A, S725A, S757A, S830A, S627D, and 4P. For FRAP, cells were transfected only with HURP WT or HURP S627A construct. For immunofluorescence, immunoprecipitations, or FRAP experiments, JetPRIME (Polyplus) transfection reagent was used to transfect HeLa Kyoto cells with the above constructs, according to the manufacturer’s protocol. For photoactivation, cells were transfected with PA-GFP-HURP and mCherry-tubulin or PA-GFP-tubulin and mCherry-H2B. In this case, cells were transfected with Fugene HD (Promega) according to manufacturer’s protocol with the following modifications: medium volume was 2 mL, DNA/Fugene ratio was 1:3 and in co-transfection, 1.25 μg of DNA was used for each plasmid.

### Immunofluorescence staining, image acquisition and analysis

For immunofluorescence staining, cells were grown on No.1.5 glass coverslips, fixed in 4% paraformaldehyde/PHEM (60 mM PIPES, 25 mM HEPES, 10 mM EGTA, 2 mM MgCl_2_) pH 6.9 for 12 min at 37°C and then permeabilized in PBS/0.1% v/v Triton X-100 pH 7.4 for 5 min at room temperature (RT). Fixed samples were blocked in PBS/5% w/v BSA pH 7.4 for 20 min at RT and incubated with the following primary antibodies of interest: anti-α-tubulin mouse monoclonal antibody (1:1,000; sc32293, Santa Cruz Biotechnology) and anti-centromere human polyclonal antibody (ACA) (1:200; 15-235, Antibodies Incorporated, Davis, CA, United States) for 1 h at RT. Cells were washed in PBS pH 7.4, incubated with the appropriate secondary antibodies conjugated either with CF^®^568 (1:1,000; 20,100, Biotium, Fremont, CA, United States) or CF^®^647 (1:1,000; 20,280, Biotium, Fremont, CA, United States) dyes for 30 min at RT and DNA was counterstained with Hoechst-33342 (10 μg/mL; Biotium). After final washes, coverslips were mounted in Mowiol 4-88 mounting medium (Applichem).

For Kif5B immunofluorescence staining, cells first were pre-extracted with BRB80-0.05% digitonin (Sigma) for 1 min, fixed in BRB80-PFA 4% for 15 min at 37°C, blocked in PBS/5% w/v BSA pH 7.4 for 20 min at RT and then incubated with anti-Kif5B rabbit polyclonal antibody (1:100; ab167429, Abcam, Cambridge, United Kingdom) for 1 h at RT. Then cells were washed in PBS pH 7.4, incubated with the appropriate secondary antibody conjugated with CF^®^488A (1:1,000; 20,012, Biotium, Fremont, CA, United States) dye for 30 min at RT and DNA was counterstained with Hoechst-33342 (10 μg/mL; Biotium). After final washes, coverslips were mounted in Mowiol 4-88 mounting medium (Applichem).

Imaging was performed on a customized Andor Revolution Spinning Disk Confocal system (Yokogawa CSU-X1; Yokogawa, Tokyo, Japan) built around an Olympus IX81 (Olympus Shinjuku, Tokyo, Japan) with 100 × 1.4NA (UPlanSApo; Olympus Shinjuku, Tokyo, Japan) or 60 × 1.42NA (UPlanXApo; Olympus Shinjuku, Tokyo, Japan) oil lenses and two digital cameras (Andor Ixon+885 or Andor Zyla 4.2 sCMOS; Andor Technology Ltd., Belfast, Northern Ireland). System was controlled by Andor IQ2 or IQ3.6 software (Andor Technology) (Bioimaging Facility, MBG-DUTH). Images were acquired as z-stacks with selected optical sections every 0.1, 0.3, 0.5 or 1 μm through the entire cell volume, according to experimental needs.

Image analysis was performed with ImageJ (National Institute of Health, United States). Pole-to-pole intensity profiles were measured in the average z-projected images. Briefly, background was subtracted and a line of 2 μm width in physical units was drawn from pole-to-pole in the channel corresponding to tubulin. Intensity plot profiles of both tubulin and HURP were measured. To account for different spindle sizes, normalized plot profiles were interpolated using GraphPad Prism 8 (GraphPad Software, La Jolla California United States). Spindle length represent the pole-to-pole distance in the average z-projected images. To measure inter-kinetochore distances, first raw image volumes were deconvolved using Huygens Professional (Scientific Volume Imaging, Netherlands, http://svi.nl) and then, distances were measured in the processed volumetric images with ImageJ. All immunofluorescence images presented here, are the maximum projection across z-dimension, in which, background has been subtracted.

### Live cell imaging and analysis

For photoactivation experiments, cells were transfected 22.5 h before imaging. Sixteen hours post-transfection cells treated with 50 ng/mL Nocodazole for 6 h, after thorough washing with PBS (1-3 times) cells were released in phenol red-free imaging medium (GIBCO) containing 10 μM MG132 (Calbiochem) and imaged 30 min post release, for up to 1.5 h at 37°C, in an environmental chamber with 5% CO_2_.

Photoactivation were performed using a Zeiss LSM780, Laser Scanning Confocal Microscope (ALMF-EMBL) controlled by Zen Black 2010 software using 63x/1.40 NA oil immersion objective or × 20/0.8 NA air objective with the pinhole wide-open. Photoactivation was stimulated in a zone perpendicular to the spindle long axis using stripes of 2 μm wide using a 405 nm laser. Pre- and post-photoactivation time frames were acquired with 488 nm laser for PA-GFP and 561 nm laser for mCherry, with intervals in the range of 1 s–8 s. Image analysis was performed with ImageJ (National Institute of Health, United States). Initially, spindle position at each frame was registered using StackReg plug-in (RigidBody option) to correct for spindle movement and the pole-to-pole intensity plot profiles were made, as previously described (Uteng et al., 2008). For cells expressing PA-GFP-tubulin, activated PA-GFP-tubulin signal was used as a binary mask, to quantify fluorescence signal. To determine the position of the center of the distribution, plot profiles were fitted with Gaussian function using MATLAB’s (R2015b) curve fitting tool (The MathWorks, Inc. Natick MA, United States) and then the position of the Gauss center versus time plots were created. The position of the Gauss center vs. time plots was non-linear and they were fitted with a second order polynomial to estimate the acceleration a (change of the slope), initial velocity $u_{0}$ and to generate a trendline. In the case of PA-GFP-HURP, the velocity extracted from the fitting process was negatively correlated with the distance of the photoactivation area from the chromosomes (Supplementary Figure S1B), due to the slight drift of cells during photoactivation. To be able to average the velocities and compare it with MT flux we calculated the velocity at a reference point. i.e., at the edge of the chromosomes (Supplementary Figure S1C). We measured the distance, $x_{0}$ , of the center of the photoactivation area from the edge of chromosomes (defined by the gap of MT staining), for each cell, and equations of motion were created:

$$\begin{array}{c} x=x_{0}+u_{0}t+\frac{1}{2}at^{2} \end{array}$$ (1.1)

$$\begin{array}{c} u_{c}=u_{0}+at \end{array}$$ (1.2)

Supposing that the edge of the chromosomes is at *x* = 0, by solving Eq. 1.1 for time, t, in which x=0 and substituting t in Eq. 1.2, the theoretical velocity, $u_{c}$ , of molecules at the edge of chromosomes was calculated (Supplementary Figure S1D). The same procedure for MT flux was followed and thus the average velocities were measured and compared in the same reference point (Supplementary Figure S1D). For analyzing cells photoactivated at the pole, the same procedure was followed except that $x_{0}$ corresponds to the distance of the photoactivation area from the pole. Statistical analysis was performed using Mann-Whitney two-tailed test in GraphPad Prism 8 (GraphPad Software, La Jolla California United States). Spearman coefficients and linear fitting were performed in GraphPad Prism 8.

For FRAP experiments, cells were transfected 22.5 h before imaging. Sixteen hours post-transfection cells treated with 50 ng/mL Nocodazole and 100 nM SiR-tubulin (Spirochrome, sc002) for 6 h, after thorough washing with PBS (1-3 times) cells were released in phenol red-free imaging medium (GIBCO) containing 10 μM MG132 (Calbiochem) or 10 μM MG132 and 10 nM Nocodazole (Sigma), and imaged 30 min post release, for up to 1.5 h at 37°C, in an environmental chamber with 5% CO_2_.

FRAP was performed with FRAPPA module (Andor Technology, Belfast, Northern Ireland) mounted on a customized Andor Revolution Spinning Disk Confocal system (Yokogawa CSU-X1) built around an Olympus IX81, with 60x/1.42 NA oil immersion objective and a digital camera (Andor iXon Ultra 897 EMCCD; Andor Technology Ltd., Belfast, Northern Ireland). The system was controlled by Andor IQ3.6 software (Bioimaging Facility, MBG-DUTH). Half of the spindle was selected with a polygon selection and was photobleached using the 488 nm laser line. Post photobleaching images were acquired using the 488 nm and the 638 nm laser lines with intervals between frames of 0.5 or 5 s for a total of 120 s. At least one frame before photobleaching was acquired for normalization purposes. Images were analyzed in ImageJ. Briefly, spindle positions were registered with ImageJ’s plugin StackReg (RigidBody option) using the tubulin channel as reference to correct spindle movements during acquisition. Afterwards, the tubulin signal at each frame was used to create a mask, segment spindle in different compartments and quantify HURP fluorescence over the spindle. The mean fluorescence intensity of whole spindle, bleached half spindle, unbleached half spindle, chromosome zone, intermediate zone and pole zone as well as background intensity were measured. Data normalization was performed in MATLAB. Briefly, background signal was subtracted before double–normalization Eq. (2.1), followed by full-scale normalization for the bleached half spindle Eq. (2.2).

$$I_{roi\_norm}\left(t\right)=\frac{I_{ref}\left(t_{pre}\right)}{I_{ref}\left(t\right)}\frac{I_{roi}\left(t\right)}{I_{roi}\left(t_{pre}\right)}$$ (2.1)

$$I_{bleached}\left(t\right)=\frac{I_{bleached\_norm}\left(t\right)-I_{bleached\_norm}\left(t_{0}\right)}{1-I_{bleached\_norm}\left(t_{0}\right)}$$ (2.2)

“Roi” either represents the unbleached or bleached compartments, “ref” is the whole spindle, “t_pre_” represents the pre-bleaching frame and “t_0_” the first post-bleaching frame. Normalized recovery curves were fitted using one-phase association or decay equations in GraphPad Prism 8.

Spindle length, mean intensity, and plot profiles of HURP and tubulin, were measured using the frames acquired pre-photobleaching.

### Mitotic cell extract, immunoprecipitation and Western blot analysis

HeLa Kyoto cells were arrested in mitosis with 100 ng/mL Nocodazole for 16 h. Mitotic cells were shaken off, centrifuged at 500 *g* for 5 min. Cells were extracted in RIPA buffer (50 mM Tris pH 8, 150 mM NaCl, 50 mM sodium orthovanadate, 10 mM sodium fluoride, 0.1% v/v NP-40, 1 mM PMSF) supplemented with complete protease inhibitors cocktail (Roche, Basel, CH) for 30 min at 4°C and centrifuged at 17,900 g for 30 min at 4°C. The concentration of the mitotic cell extracts was determined by the Bradford assay (Bio-Rad, Hercules, CA, United States).

For immunoprecipitations (1 mg of mitotic extract per IP) anti-HURP rabbit polyclonal antibody (ab70744, Abcam, Cambridge, United Kingdom), anti-HURP (GKAP) rabbit polyclonal antisera, anti-HURP (DHL5) rabbit polyclonal antisera (Koffa et al., 2006) or anti-GFP mouse monoclonal antibody (11814460001, Roche, Basel, CH) were used. For the co-IP with the HURP antibodies, pre-immune IgG, rabbit IgG or anti-GFP rabbit polyclonal antibody (ab290, Abcam, Cambridge, United Kingdom) were used as a control. To reduce the amount of nonspecific protein binding, the supernatant was first incubated with the protein A agarose beads (MilliporeSigma Upstate, Burlington, MA, United States) for 1 h at 4°C. After centrifugation, the supernatant was incubated with the antibody for 6 h at 4°C. Post incubation, fresh protein A agarose beads (MilliporeSigma Upstate, Burlington, MA, United States) were added to the reaction and incubated at 4°C overnight. As for the co-IP with the GFP antibody, the mitotic extracts were incubated with the antibody for 6 h at 4°C. After the end of incubation, fresh protein G agarose beads (MilliporeSigma Upstate, Burlington, MA, United States) were added to the reaction and incubated at 4°C overnight.

Next, in both cases beads were collected by centrifugation and washed five times in RIPA buffer. Finally, bound complexes were boiled, centrifuged, subjected to SDS-PAGE and analyzed by Western blot.

For Western blot analysis, the protein extracts were loaded on SDS-PAGE, transferred to a nitrocellulose membrane and probed with anti-IAK1/Aurora A mouse monoclonal antibody (1:1,000; 610,938, BD Transduction Laboratories, NULL, United States), anti-phospho-Aurora A rabbit monoclonal antibody (Thr288) (1:1,000; 3079S, Cell Signaling, Danvers, MA, United States), anti-HURP rabbit polyclonal antisera (DHL5) [1:1,000 (Koffa et al., 2006)], anti-TPX2 rabbit polyclonal antisera [1:1,000 (Gruss et al., 2002)], anti-Eg5 rabbit whole antisera (1:1,000; NB500-181, Novus Biologicals, Littleton, CO, United States), anti-Importin *ß* mouse monoclonal antibody (1:1,000; ab2811, Abcam, Cambridge, United Kingdom), anti-GFP rabbit polyclonal antibody (1:1,000; ab290, Abcam, Cambridge, United Kingdom) anti-Dynein rabbit polyclonal antibody [1:2,000 (Trokter et al., 2012)] and anti-Kif5B rabbit monoclonal antibody (1:1,000; ab167429, Abcam, Cambridge, United Kingdom) or anti-Kif5B mouse monoclonal antibody (1:1,000; sc-133184, Santa-Cruz biotechnology).

### Mass spectrometry

Samples for mass spectrometry were separated on 4%–12% gradient NuPAGE gels (Invitrogen, Carlsbad, CA, United States), followed by colloidal Coomassie blue staining. Gel lanes were cut into 14 slices and then digested with trypsin. Peptides were separated using the Proxeon EasyNanoLC system (Thermo Fisher, Waltham, MA, United States) fitted with a trapping column (Hydro-RP C18 (Phenomenex, Torrance, CA, United States), 100 μm × 2.5 cm, 4 μm) and an analytical column (Reprosil C18, 75 μm × 15 cm, 3 μm, 100 Å). The outlet of the analytical column was coupled directly to an HCT Ultra Ion Trap mass spectrometer (Bruker Daltonics, Billerica, MA, United States) using the ESI nanoflow source in positive ion mode. Peptides were identified via Mascot (Matrix Science, London, United Kingdom) using the Swiss-Prot database.

### Statistical analysis

All statistical analysis has been performed in GraphPad Prism 8 using the two-tailed Mann-Whitney test. In Supplementary Figure S1D the Spearman correlation method in GraphPad prims 8 was used. For all figures: *p≤0.05 , **p≤0.01 , ***p≤0.001 , ****p≤0.0001 .

## Results

### MT flux alterations affect HURP dynamics over metaphase spindle

**FIGURE 1:**
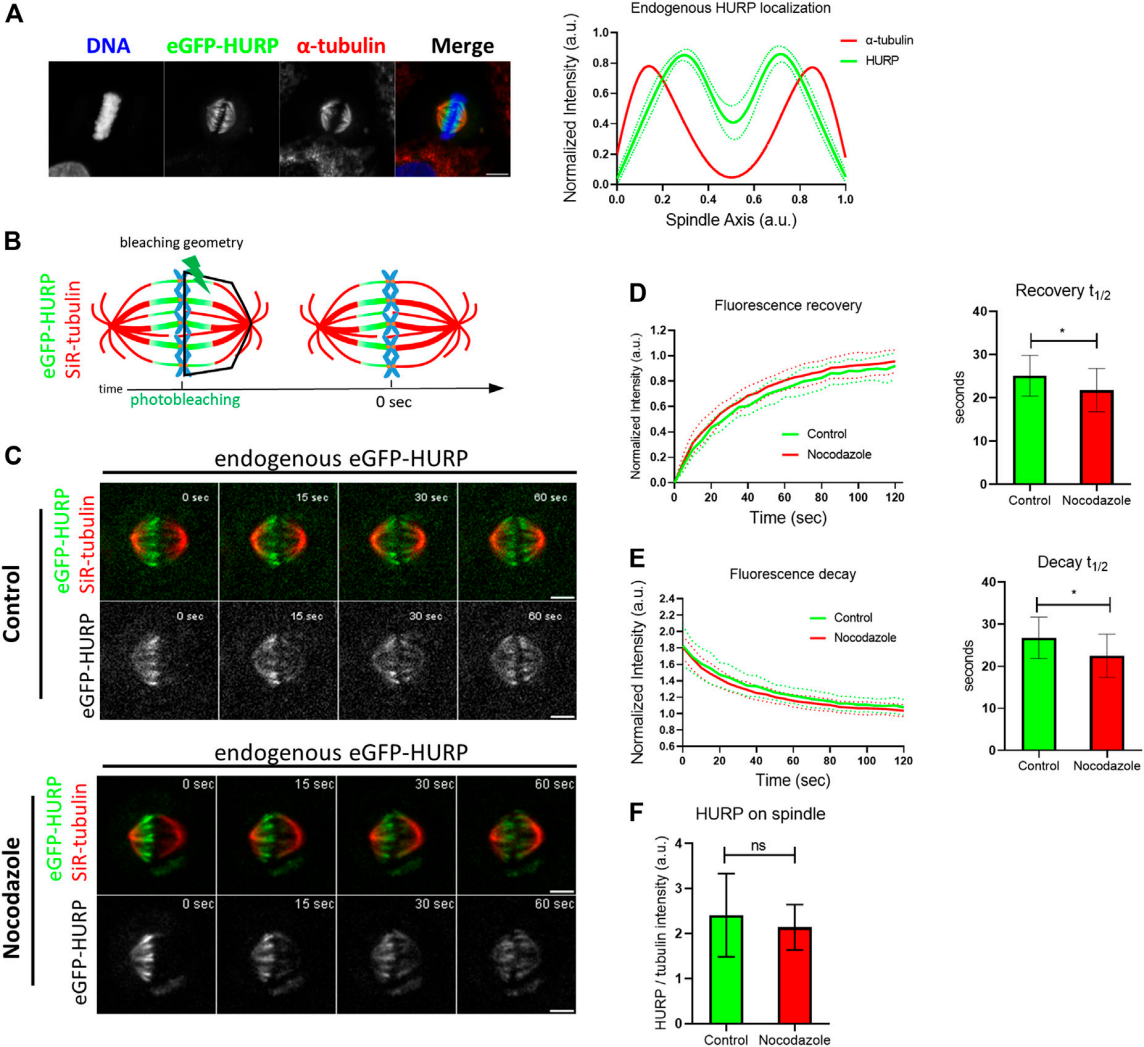
Endogenous HURP dynamics after photobleaching half of the mitotic spindle. **(A)** Left: Representative image of HeLa Kyoto cell endogenously expressing eGFP-HURP, arrested in metaphase. Immunofluorescence was performed against *α*-tubulin and DNA was counterstained with Hoechst. Right: Endogenous eGFP-HURP and *α*-tubulin intensity plot profiles along HeLa Kyoto metaphase spindle long axis. Bold-lines indicate the average of *n* = 16 cells, and the green dashed-lines indicate the 95% CI. **(B)** Schematic representation of a FRAP experiment performed on HeLa Kyoto cells endogenously expressing eGFP-HURP, counterstained with SiR-tubulin and arrested in metaphase. Tubulin signal was used as a spatial reference for drawing a polygon (bleaching geometry) to photobleach HURP molecules. Time-point 0 s represents the first acquired frame post photobleaching. **(C)** Representative images of control (upper panel) and Nocodazole (lower panel) treated HeLa Kyoto cells at different timepoints post photobleaching. **(D)** Left: Fluorescence recovery curves of eGFP-HURP in control and Nocodazole treated cells. Bold-lines indicate the mean and dashed-lines represent the ± S.D. Right: One-phase exponential fitting resulted in half-lives of $t_{1/2}=25\;\pm 5\;\sec$ for control cells, (*n* = 18 cells) and $t_{1/2}=22\;\pm 5\;\sec$ for Nocodazole treated cells (*n* = 20 cells) (mean ± S.D.; *p = 0.032). **(E)** Left: eGFP-HURP fluorescence decay curves of the unbleached spindle half in control and Nocodazole treated cells. Bold-lines indicate the mean and dashed-lines represent the ±S.D. Right: One-phase decay fitting resulted in half-lives of $t_{1/2}=27\;\pm 5\;\sec$ for control, (*n* = 18 cells) and $t_{1/2}=23\;\pm 5\;\sec$ for Nocodazole treated cells (*n* = 20 cells). (mean ± S.D.; *p = 0.016). **(F)** Spindle-bound endogenous eGFP-HURP quantification (relative to SiR-tubulin signal), derived from the pre-photobleached frames. (control, *n* = 23 cells; Nocodazole, *n* = 30 cells). Scale bar denotes 5 μm.

In order to investigate HURP dynamics on the metaphase spindle, the CRISPR/Cas9 system was used to generate HeLa Kyoto cells endogenously expressing eGFP-HURP. Proper localization of the eGFP-tagged HURP expressed at endogenous levels was confirmed by immunofluorescence analysis in metaphase-arrested cells near chromosomes (Figure 1A).

Fluorescence Recovery After Photobleaching (FRAP) was employed to study HURP dynamics. HeLa Kyoto cells endogenously expressing eGFP-HURP were synchronized, maintained in metaphase using the proteasome inhibitor MG132 and stained with SiR-tubulin. Using tubulin signal as a spatial reference, eGFP-HURP was photobleached on half of the mitotic spindle (Figure 1B). Fluorescence recovery of eGFP-HURP was monitored for up to 120 s, with a 5-s interval, in both control cells and cells treated with 10 nM of Nocodazole as a MT flux suppressing agent (Vasquez et al., 1997; Ma et al., 2010; Rajendraprasad et al., 2021) (Figure 1C).

Fluorescence recovery half-lives were calculated from the FRAP data points which were normalized and fitted with one-phase exponential equation (Figure 1D). In control cells, a mean fluorescence recovery half-life of $t_{1/2}=25\;\pm 5\;\sec$ (*n* = 18 cells) was measured, while in Nocodazole-treated cells a slightly faster, but statistically different mean half-life of $t_{1/2}=22\;\pm 5\;\sec$ (n = 20 cells) was calculated (Figure 1D). To further understand the increased HURP dynamics on stabilized MTs, we also measured and quantified HURP fluorescence decay on the unbleached spindle half, by fitting a one-phase decay equation to the normalized data points. In control cells, fluorescence decay was calculated with a half-life of $t_{1/2}=27\;\pm 5\;\sec$ (*n* = 18 cells). In Nocodazole-treated cells, a half-life of $t_{1/2}=23\;\pm 5\;\sec$ (*n* = 20 cells) was calculated, also significantly different from the control (Figure 1E).

No significant difference on spindle-bound HURP was observed between control and Nocodazole-treated cells (Figure 1F). In addition, neither spindle length nor HURP localization were altered upon Nocodazole treatment (Supplementary Figure S2A; Supplementary Figure S2B). These results suggest that MT flux suppression and MT stabilization affect HURP dynamics, by increasing the protein’s MT off-rate.

### MT flux alterations increase HURP off-rate in the chromosome’s vicinity

**FIGURE 2:**
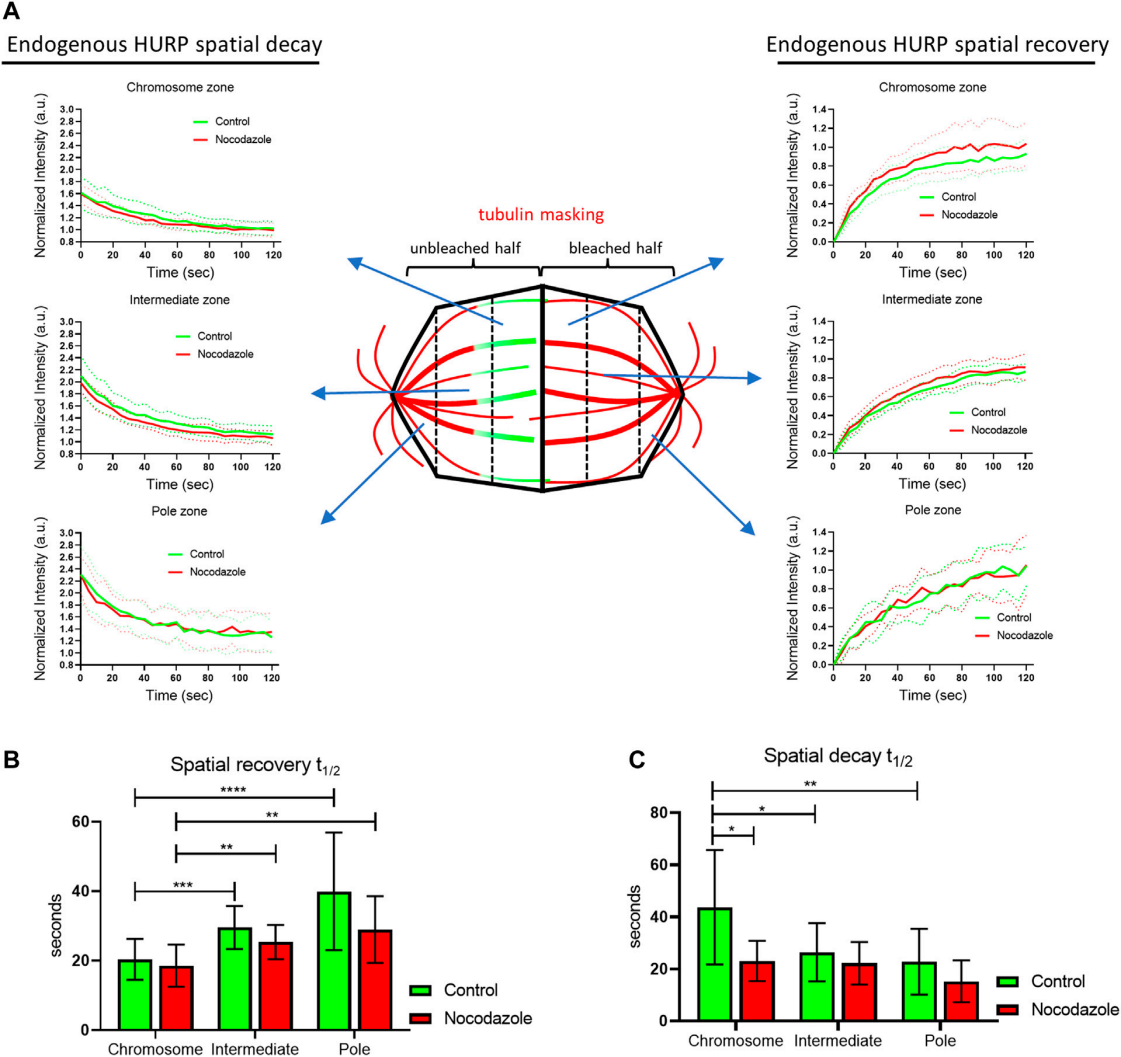
Endogenous HURP spatial dynamics after photobleaching half of the mitotic spindle. **(A)** Schematic representation of spatial dynamics analysis. SiR-tubulin signal was used as a mask for metaphase spindle compartmentalization in six regions. The fluorescence decay curves of the unbleached spindle half and the fluorescence recovery curves of the bleached spindle half were generated. Bold-lines indicate the mean value and dashed-lines represent the ± S.D. **(B)** Fluorescence recovery half-lives were quantified by one-phase exponential fitting. (control, *n* = 16 cells; Nocodazole, *n* = 13 cells). Bars represent the mean value ± S.D. **(C)** Fluorescence decay half-lives were quantified by one-phase decay fitting. (control, *n* = 17 cells; Nocodazole, *n* = 13 cells). Bars represent the mean value ±S.D.

To further understand how MTs’ stabilization affects HURP dynamics, eGFP-HURP spatial recovery and decay were calculated after photobleaching, by compartmentalizing the mitotic spindle into six zones (pole, intermediate, and chromosome zones in both the unbleached and bleached halves of the spindle), using tubulin signal as a mask. HURP fluorescence in each zone was measured over time (Figure 2A).

In control cells, curve fitting of FRAP data showed that HURP fluorescence recovered significantly faster in the chromosome zone with a mean half-life of $t_{1/2}=20\;\pm 6\;\sec$ (*n* = 16 cells), compared to the intermediate zone ( $t_{1/2}=30\;\pm 6\;\sec$ ; *n* = 16 cells), or the pole zone ( $t_{1/2}=40\;\pm 17\;\sec$ ; *n* = 16 cells) (Figure 2B). The same fluorescence recovery pattern was observed in cells treated with a low dose of Nocodazole. In these cells, the fluorescence of HURP recovered significantly faster at the chromosome zone ( $t_{1/2}=19\;\pm 6\;\sec$ ; *n* = 13 cells) compared to intermediate zone ( $t_{1/2}=25\;\pm 5\;\sec$ ; *n* = 13 cells) or the pole zone $\left(t_{1/2}=29\;\pm 10\;\sec\right.$ ; *n* = 13 cells) (Figure 2B). These results show that HURP molecules preferentially bind to spindle MTs in the vicinity of the chromosomes, consistent with the Ran-GTP model, regardless of MTs dynamics.

Interestingly, when comparing the spatial recovery rates of HURP fluorescence between control and Nocodazole-treated cells for each compartmentalized zone, no significant difference was observed, most likely due to the small size of the segmented zones.

Nevertheless, when the same compartmentalized analysis was applied to spatial HURP fluorescence decay rates, Nocodazole-treatment significantly decreased HURP fluorescence decay half-life in the chromosome zone to $t_{1/2}=23\;\pm 8\;\sec$ (*n* = 13 cells), compared to control cells ( $t_{1/2}=44\;\pm 22\;\sec$ ; *n* = 17 cells). No other significant difference in the decay half-lives was observed in the intermediate zone ( $t_{1/2}=22\;\pm 8\;\sec$ ; *n* = 13 cells, compared to $t_{1/2}=26\;\pm 11\;\sec$ ; *n* = 17 cells) or pole zone ( $t_{1/2}=15\;\pm 8\;\sec$ ; *n* = 13 cells, compared to $t_{1/2}=23\;\pm 13\;\sec$ ; *n* = 17 cells) between Nocodazole-treated cells and control (Figure 2C).

These results suggest that low doze Nocodazole treatment, which affects MT flux, increases the off-rate of HURP molecules from the chromosome zone, without altering their chromosome proximal distribution.

### Poleward movement of HURP molecules photoactivated at the chromosome zone

**FIGURE 3:**
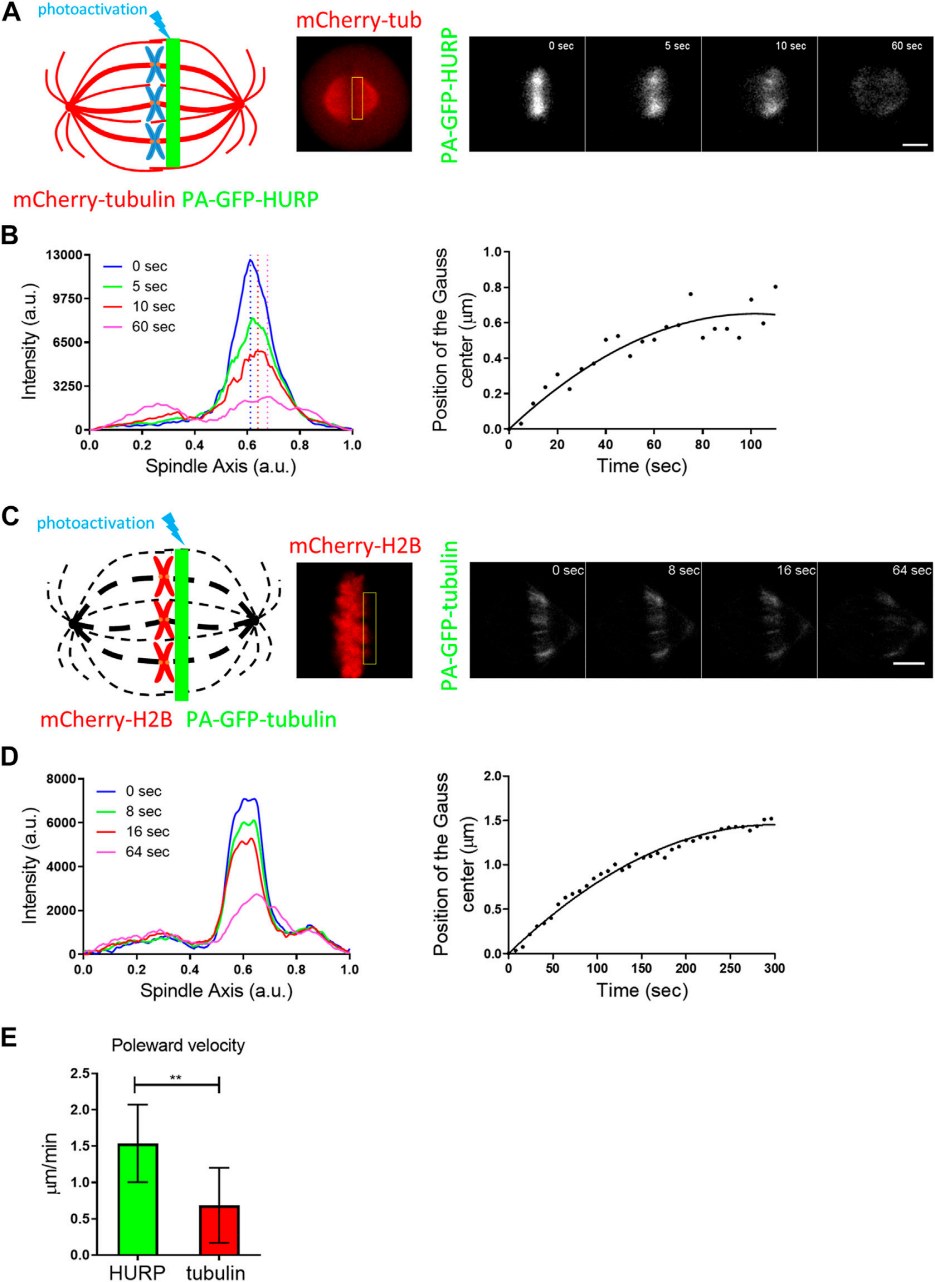
PA-GFP-HURP molecules photoactivated at the chromosome zone **(A)** Left: Schematic representation of photoactivation experiments in cells co-expressing PA-GFP-HURP and mCherry-tubulin in HeLa Kyoto cells. A 2 μm-wide area perpendicular to the spindle long axis (green stripe) was used to photoactivate PA-GFP-HURP molecules. Middle: mCherry-tubulin signal was used as a spatial reference to photoactivate PA-GFP-HURP molecules at the chromosome zone and register moving spindles during data processing. Right: Representative images of PA-GFP-HURP molecules at different timepoints post photoactivation. Scale bar denotes 5 μm. **(B)** Left: Representative pole to pole fluorescence intensity profiles showing the distribution of the photoactivated PA-GFP-HURP molecules near the chromosomes, at different timepoints post photoactivation. The t=0 sec represents the first timepoint post photoactivation. The vertical dashed-lines designate the center of fluorescence distribution of photoactivated PA-GFP-HURP molecules at the corresponding timepoints. Right: Representative plot showing the center of fluorescence distribution over time, fitted by a second order polynomial to calculate PA-GFP-HURP poleward velocity. **(C)** Left: Schematic representation of photoactivation experiments in cells co-expressing PA-GFP-tubulin and mCherry-H2B, in HeLa Kyoto cells. A 2 μm-wide area perpendicular to the spindle long axis (green stripe) was used to photoactivate PA-GFP-tubulin molecules. Dark dashed-lines represent the expected mitotic spindle shape. Middle: mCherry-H2B signal was used as a spatial reference in order to photoactivate PA-GFP-tubulin at the chromosome zone, as well as to register cell movements during data processing. Right: Representative images of PA-GFP-tubulin at different timepoints post photoactivation. Scale bar denotes 5 μm. **(D)** Left: Representative pole to pole fluorescence intensity profiles showing the distribution of the photoactivated PA-GFP-tubulin molecules near the chromosomes, at different timepoints post photoactivation. The t=0 sec represents the first timepoint post photoactivation. Right: Representative plot showing the center of fluorescence distribution over time, fitted by a second order polynomial to calculate PA-GFP-tubulin poleward velocity (MT flux). **(E)** Comparison of PA-GFP-tubulin and PA-GFP-HURP poleward velocities. Values indicate the mean ± S.D. (PA-GFP-tubulin, *n* = 7 cells; PA-GFP-HURP, *n* = 14 cells; **p = 0.0042).

To gain further insight into the binding kinetics of HURP molecules on the metaphase spindle, we performed photoactivation experiments. HeLa Kyoto cells were co-transfected with PA-GFP-HURP and mCherry-tubulin, synchronized and arrested in metaphase using the proteasome inhibitor MG132. Initially, PA-GFP-HURP molecules were photoactivated in a 2 μm-wide stripe near the chromosomes, perpendicular to spindle long axis, using the mCherry-tubulin signal as a spatial reference. Post-activation frames were acquired for up to 110 s (Figure 3A).

To follow the movement of the photoactivated molecules over time, a Gaussian curve was fitted to each fluorescence intensity profile, and the center of the fluorescence distribution was determined (Figure 3B left). By fitting the distributions’ centers over time with a second order polynomial (Figure 3B right), we observed a movement of photoactivated HURP molecules towards the spindle pole at an average rate of $u_{poleward}=1.53\pm 0.53\mu m/\min$ (*n* = 14 cells).

Since HURP binds to spindle MTs, the observed poleward movement could be attributed to MT flux. To test this hypothesis, the same photoactivation protocol was performed in HeLa Kyoto cells co-transfected with mCherry-H2B and PA-GFP-tubulin and MT flux was measured (Figure 3C). Following the same analysis procedure applied to PA-GFP-HURP, an average rate of $u_{flux}=0.69\pm 0.51\mu m/\min$ (*n* = 7 cells) was measured (Figure 3D). Interestingly, the poleward velocity of photoactivated HURP molecules was significantly higher than MT flux (Figure 3E). These findings suggest that the observed poleward movement of HURP molecules cannot be simply attributed to MT flux.

### Equatorward movement of HURP molecules photoactivated near the poles

**FIGURE 4:**
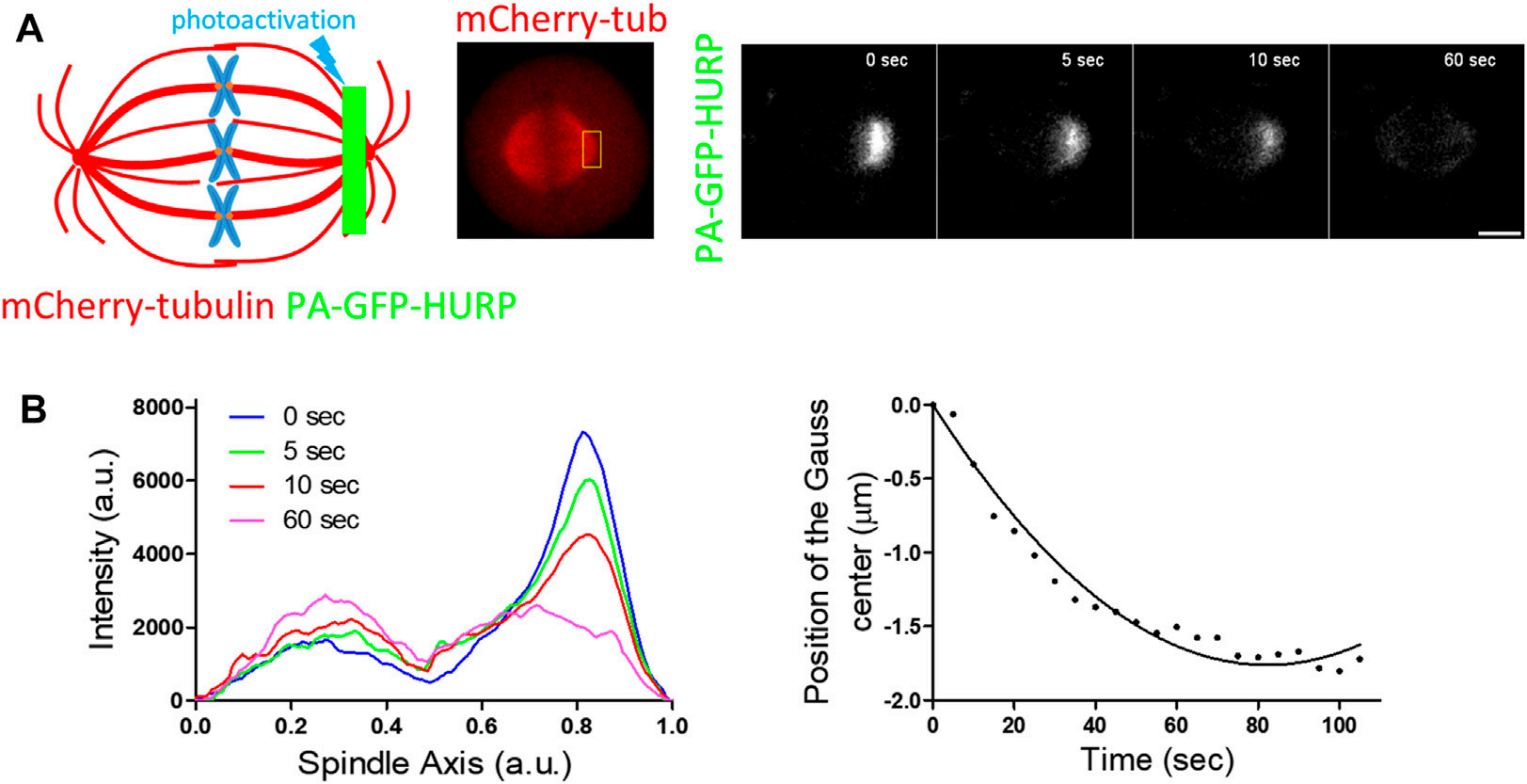
PA-GFP-HURP molecules photoactivated at the pole zone **(A)** Left: Schematic representation of photoactivation experiments in cells co-expressing PA-GFP-HURP and mCherry-tubulin. A 2 μm-wide area perpendicular to the spindle long axis (green stripe) was used to photoactivate PA-GFP-HURP molecules. Middle: mCherry-tubulin signal was used as a spatial reference to photoactivate PA-GFP-HURP molecules at the pole zone and register moving spindles during data processing. Right: Representative images PA-GFP-HURP molecules at different timepoints post photoactivation. Scale bar denotes 5 μm. **(B)** Left: Representative pole to pole fluorescence intensity profiles showing the distribution of the photoactivated PA-GFP-HURP molecules near the poles at different timepoints post photoactivation. The t=0 sec represents the first timepoint post photoactivation. Right: Representative plot showing the center of fluorescence distribution over time, fitted by a second order polynomial to calculate PA-GFP-HURP poleward velocity.

Next, PA-GFP-HURP molecules expressed in HeLa Kyoto mitotic cells, co-expressing mCherry-tubulin, were photoactivated at the pole zone (Figure 4A). Data analysis showed that photoactivated HURP molecules move towards the chromosomes (Figure 4B) at an average rate of $u_{equatorward}=2.94\pm 0.42\;\mu m/\min$ (*n* = 5 cells). One possible explanation for this movement is the association of HURP with a kinesin motor protein, which could transport it towards the plus-ends of MTs. Alternatively, HURP molecules could fall off from the minus-ends of MTs and re-load at their plus-ends.

### Identification of HURP mitotic partners in mammalian cells

The above photoactivation experiments revealed distinct dynamics of HURP at the equator and the pole of a metaphase spindle. HURP molecules photoactivated near the chromosomes moved poleward, faster than MT flux, whereas HURP molecules photoactivated near the pole, moved towards the metaphase equator. If HURP was simply loaded onto MT plus-ends due to its release from the inhibitory binding of Importins, moving towards the minus-end due to MT flux, and then randomly falling off from the MTs, we would observe the protein moving only poleward.

HURP has been reported to interact with several partners including TPX2, Importin β, Aurora A and, Eg5 in *Xenopus* egg extracts (Koffa et al., 2006). Therefore, the different dynamics of HURP at the two distinct sites of the metaphase spindle could be attributed to the presence of different HURP-bound complexes.

**FIGURE 5:**
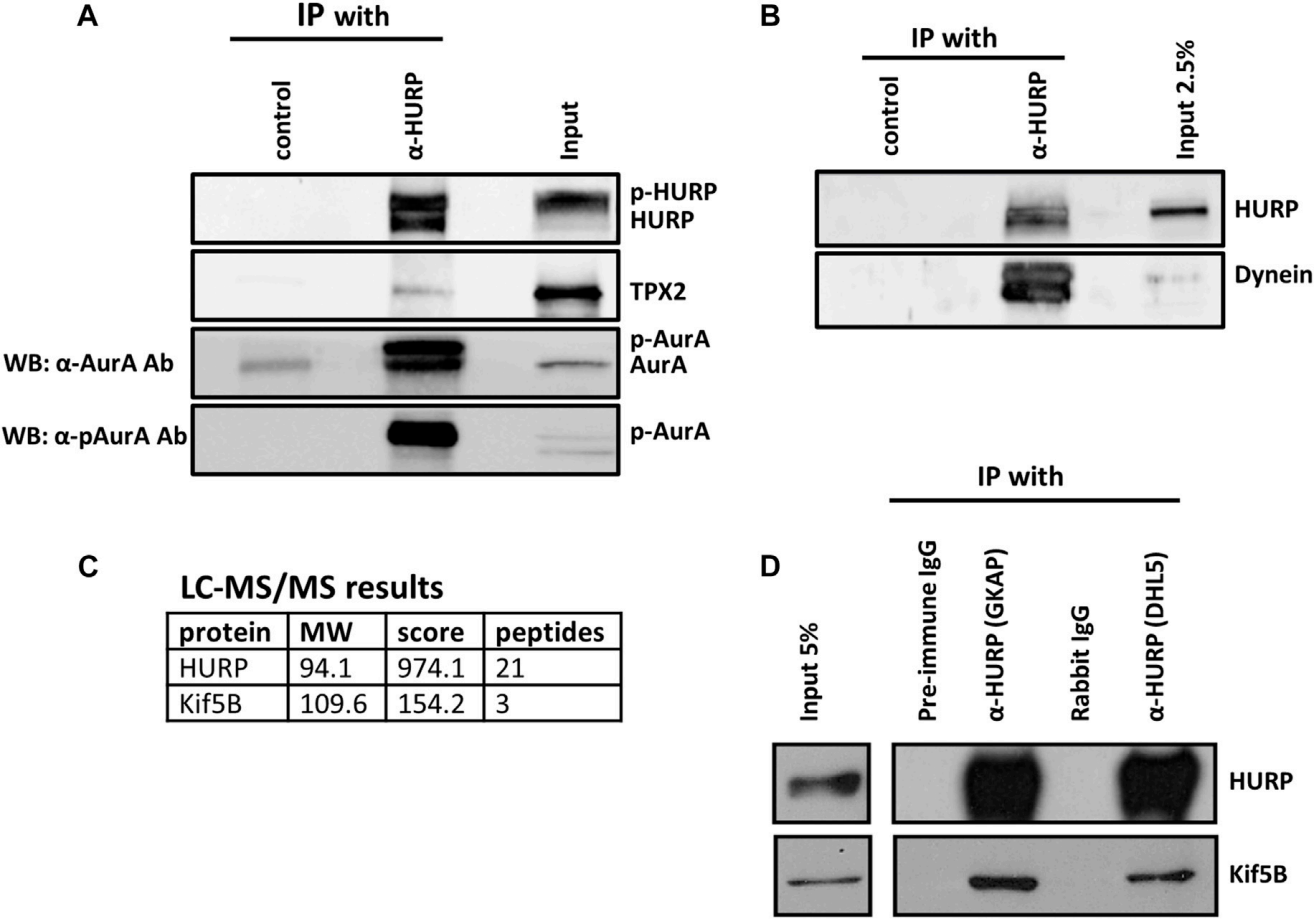
HURP interacting partners during mitosis **(A)** Co-immunoprecipitation assays using an antibody against HURP in HeLa Kyoto mitotic cell extracts. Rabbit IgG or anti-GFP rabbit polyclonal antibody were used as the respective controls. HURP and bound complexes were analyzed by Western blot using antibodies against HURP, TPX2 and two antibodies against Aurora A, as indicated on the left side (α-AurA antibody, or *α*-pAurA antibody). Input represents 6% of the total amount of protein used for the immunoprecipitation assay for HURP and p-Aurora A, 2% for TPX2, 5% for Aurora A. **(B)** Co-immunoprecipitation assay using an antibody against HURP in HeLa Kyoto mitotic cell extracts. Anti-GFP rabbit polyclonal antibody was used as a control. HURP and bound complexes were analyzed by Western blot using antibodies against HURP and Dynein. Input represents 2.5% of the total amount of protein used for the immunoprecipitation assay. **(C)** HeLa Kyoto mitotic extracts were immunoprecipitated using an antibody against HURP. The isolated complexes were analyzed by LC-MS/MS. Kif5B was identified as a possible partner of HURP. **(D)** Co-immunoprecipitation assays using either an antibody against the middle part (GKAP) or the N-terminal part of HURP (DHL5), in HeLa Kyoto mitotic cell extracts. Pre-immune IgG or Rabbit IgG were used as the respective controls. HURP and bound complexes were analyzed by Western blot using antibodies against HURP and Kif5B. Input represents 5% of the total amount of protein used for the immunoprecipitation assay.

To investigate this further, we performed co-immunoprecipitation experiments in HeLa Kyoto mitotic extracts using an antibody against HURP. Western blot analysis confirmed the co-immunoprecipitation of Aurora A and TPX2 with HURP (Figure 5A). Additionally, Eg5 was found to interact with HURP, albeit to a lesser extent (Supplementary Figure S3A).

In order to further understand the poleward movement of HURP molecules photoactivated in the chromosome zone (Figure 3), we investigated the potential interaction between HURP and Dynein. Western blot analysis of the HURP-bound complexes showed that Dynein co-immunoprecipitated with HURP (Figure 5B).

Furthermore, immunoprecipitation experiments in HeLa Kyoto mitotic extracts using antibodies against HURP were performed, followed by mass-spectrometry analysis. This approach identified Kif5B as a potential partner of HURP (Figure 5C). The interaction between HURP and Kif5B was further confirmed through additional immunoprecipitation experiments using two different antibodies against HURP (Figure 5D). Immunofluorescence analysis performed on metaphase-arrested HeLa Kyoto cells, also supported the presence of Kif5B on spindle MTs in the vicinity of chromosomes (Supplementary Figure S3B).

Based on these findings we can conclude that HURP in mammalian cells is capable of interacting with several proteins including TPX2, Aurora A, Kif5B, Dynein, and potentially Eg5. However, based on the conducted experiments, we were unable to determine whether HURP forms a single complex or multiple complexes during mitosis.

### Aurora A-dependent HURP phosphorylation at Ser627 residue is required for proper localization and recovery dynamics after photobleaching half of the mitotic spindle

**FIGURE 6:**
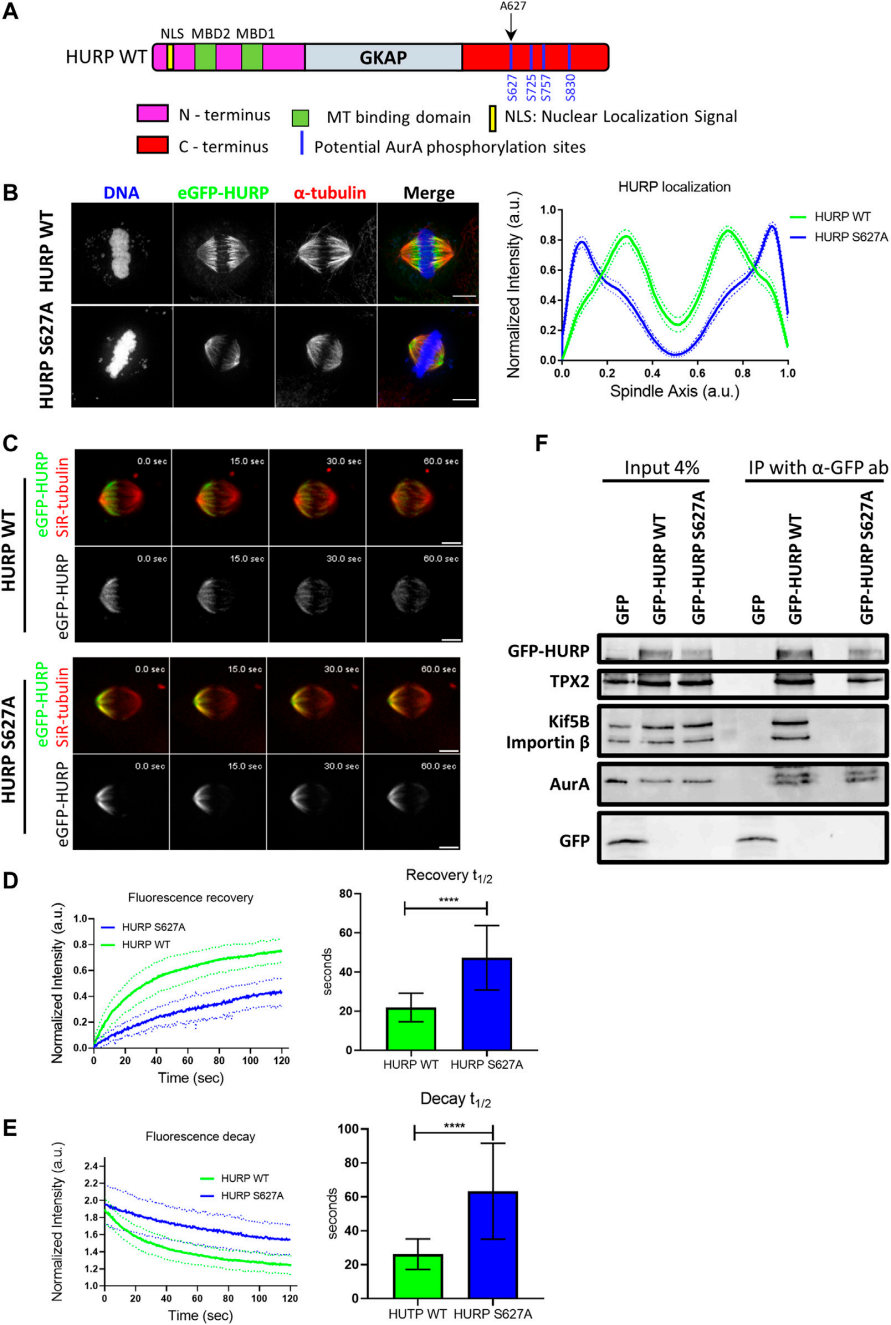
Aurora A-dependent HURP phosphorylation at the Ser627 alters HURP localization and recovery dynamics after photobleaching **(A)** Schematic representation of WT HURP. Four potential Aurora A phosphorylation sites (blue lines) are located in the C-terminus. Black arrow indicates the Serine residue that is mutated to Alanine to create the HURP S627A mutant. **(B)** Left: Representative images of HeLa Kyoto cells transfected with either eGFP-HURP WT or eGFP-HURP S627A mutant and arrested at metaphase using MG132. Immunofluorescence has been performed against *α*-tubulin. DNA was counterstained with Hoechst. Right: Intensity plot profiles along metaphase spindle long axis of HeLa Kyoto cells transfected with either eGFP-HURP WT or eGFP-HURP S627A mutant. Lines indicate the mean values whereas dots represent the ± S.D. (eGFP-HURP WT, *n* = 46 cells; eGFP-HURP S627A mutant, *n* = 69 cells). **(C)** Representative images of cells expressing eGFP-HURP WT (upper panel) or eGFP-HURP S627A (lower panel) at different timepoints post photobleaching. Scale bars denote 5 μm. **(D)** Left: Fluorescence recovery curves of eGFP-HURP. Bold-lines indicate the mean value and dashed-lines represent the ±S.D. Right: One-phase exponential fitting resulted in half-lives of $t_{1/2}=22\;\pm 7\;\sec$ for HURP WT (*n* = 17 cells), and $t_{1/2}=47\;\pm 16\;\sec$ for HURP S627A (*n* = 26 cells) (mean ± S. D.; ****p < 0.0001). **(E)** Left: eGFP-HURP fluorescence decay curves of the unbleached spindle half. Bold-lines indicate the mean value and dashed-lines represent the ± S.D. Right: One-phase decay fitting resulted in half-lives of $t_{1/2}=26\;\pm 9\;\sec$ for HURP WT (*n* = 14 cells), and $t_{1/2}=63\;\pm 28\;\sec$ for HURP S627A (*n* = 23 cells) (mean ± S. D.; ****p < 0.0001). **(F)** Co-immunoprecipitation assays using an antibody against GFP in HeLa Kyoto mitotic extracts. HeLa Kyoto cells were transfected with eGFP, eGFP- HURP WT, or eGFP-HURP S627A, and bound complexes were analyzed by Western blot using antibodies against Kif5B, TPX2, Importin *β*, Aurora A, and GFP. Input lanes represent 4% of the total amount of protein used for the immunoprecipitation assays.

HURP is a known substrate of the mitotic Aurora A kinase. Four potential Aurora A phosphorylation sites have been identified in HURP C-terminal region (Figure 6A). To investigate the impact of these phosphorylation sites on HURP localization and function, we generated dephospho-mimetic mutants of HURP, where Serine residues were replaced with Alanine at each potential phosphorylation site (S627A, S725A, S757A, S830A), as well as a phospho-null mutant containing all four-point mutations (4P), via PCR mutagenesis. Immunofluorescence analysis was performed on HeLa Kyoto cells transfected with the abovementioned HURP mutants, arrested in metaphase. Cells expressing either the eGFP-HURP S627A, or the eGFP-HURP 4P construct exhibited distinct localization pattern compared to cells expressing eGFP-HURP WT (Supplementary Figure S4A). The mutation of Serine to Alanine at residue 627 led to mis-localization of HURP, with accumulation at spindle poles instead of the chromosome vicinity, as demonstrated by the longitudinal intensity profiles (Figure 6B). Notably, only 42.3% of cells transfected with the eGFP-HURP S627A construct exhibited normal spindle formation (Supplementary Figure S4B, upper panel and right panel). However, the mis-localization of the HURP S627A mutant was not a consequence of altered spindle formation since only spindles with normal phenotypes (as determined by their MT distribution and spindle size) were included in the analysis (Supplementary Figure S4C).

Furthermore, we generated the phospho-mimetic mutant of HURP, HURP S627D, where the Serine residue at 627 was replaced with Aspartic Acid. Expression of HURP S627D disrupted spindle organization in the majority of cells (92.3%), with HURP molecules predominantly localizing in the cytoplasm (Supplementary Figure S4B, lower panel and right panel). These data provide evidence that phosphorylation of Ser627 is essential for the chromosome-proximal localization of HURP during metaphase.

To assess the impact of Ser627 phosphorylation on HURP protein dynamics, we performed FRAP experiments. Metaphase-arrested HeLa Kyoto cells expressing either the eGFP-HURP WT, or the eGFP-HURP S627A mutant were subjected to half-spindle photobleaching, and fluorescence recovery was monitored over 120 s (Figure 6C). Fluorescence recovery curves of the bleached region were created and fitted with one-phase exponential function. In control cells expressing HURP WT, a mean half-life of $t_{1/2}=22\;\pm 7\;\sec$ (*n* = 17 cells) was measured. Interestingly, the recovery of the HURP S627A mutant ( $t_{1/2}=47\;\pm 16\;\sec$ ; *n* = 26 cells) was significantly slower than that of HURP WT (Figure 6D). Moreover, the decay of HURP fluorescence was analyzed by fitting one-phase exponential decay curves. The decay of HURP WT was significantly faster ( $t_{1/2}=26\;\pm 9\;\sec$ ; *n* = 14 cells) compared to HURP S627A mutant ( $t_{1/2}=63\;\pm 28\;\sec$ ; *n* = 23 cells) (Figure 6E). The combined results of fluorescence recovery and decay, indicate a stronger binding of the dephospho-mimetic HURP S627A mutant to spindle MTs. To exclude the possibility that overexpression of HURP affects its dynamics, we also compared the fluorescence recovery after photobleaching in cells expressing eGFP-HURP either at endogenous levels, or transiently overexpressed, and found no significant differences (Supplementary Figure S5). These findings collectively suggest that phosphorylation of Ser627 plays a crucial role in both the dynamics and chromosome-proximal localization of HURP.

Moreover, we investigated whether phosphorylation of Ser627 affects the interaction of HURP with its binding partners. Co-immunoprecipitation experiments were performed using an anti-GFP antibody in mitotic extracts from HeLa Kyoto cells transfected with either eGFP, eGFP-HURP WT, or eGFP-HURP S627A. HURP WT was found to interact with Kif5B, TPX2, Importin *ß* and Aurora A (Figure 6F). In contrast, the dephospho-mimetic HURP S627A mutant exhibited an abolished interaction with Kif5B and Importin β, while displaying a similar affinity for TPX2 and Aurora A (Figure 6F). These results provide evidence for the importance of Ser627 phosphorylation in mediating the association of HURP with certain binding partners.

In conclusion, the above results highlight the critical role of Aurora A-dependent phosphorylation at the Ser627 residue of HURP in its accurate localization to the chromosome vicinity during metaphase, as well as its dynamic behavior and interaction with key mitotic proteins.

## Discussion

In this study we focused on the spatiotemporal dynamics of HURP protein on the metaphase spindle in human cells. Previous research has demonstrated that HURP molecules reposition themselves dynamically between the two spindle halves, as kt-fibers grow and shrink to align chromosomes in the metaphase plate (Castrogiovanni et al., 2022). Thus, we hypothesized that stabilizing MTs with a low dose of Nocodazole would inhibit this phenomenon and reduce HURP dynamics during metaphase. Through photobleaching half of the mitotic spindle, we observed that HURP molecules exhibited an increased recovery rate in MT-stabilized spindles, indicating increased dynamics. (Figure 1D). Further analysis revealed that this faster recovery rate resulted from an increased unbinding rate of HURP molecules from the unbleached half spindle (Figure 1E). Notably, the spatial analysis of fluorescence decay and recovery demonstrated that the unbinding rate of HURP molecules was predominantly affected in the chromosome zone of MT-stabilized metaphase spindles (Figure 2C). Considering that both HURP and low dose Nocodazole treatment stabilize MTs, we propose that the observed increase in HURP dynamics over Nocodazole-stabilized kt-fibers could act as a counter-balance mechanism, preventing MT overstabilization and restoring proper MT dynamics.

In addition, our experiments showed that HURP molecules recovered faster in the chromosome zone, compared to the intermediate and pole zones (Figure 2B). This recovery pattern is consistent with the release of HURP from inhibitory binding with Importin *ß* in the vicinity of chromosomes in a Ran-regulated manner (Nachury et al., 2001; Wiese et al., 2001; Gruss et al., 2002). This release allows HURP to bind to MTs and participate in spindle dynamics. Similar recovery pattern was observed in cells treated with Nocodazole (Figure 2B).

Furthermore, our fluorescence decay analysis indicated that HURP molecules left the pole zone faster than molecules localized near the chromosomes (Figure 2C). These findings align with a recent model proposing that the high concentration of Ran-GTP near the chromosomes releases HURP’s second Microtubule Binding Domain (MTBD) from the inhibitory binding of Importin *ß* and establishes polarized HURP localization along MTs (Tsuchiya et al., 2021). However, in Nocodazole-treated cells, HURP molecules left the chromosome zone at a similar rate to those in the intermediate zone, while maintaining proper localization (Figures 2C, Figure 1C and Supplementary Figure S2B). These results indicate that HURP binding and localization on metaphase spindle may be regulated by additional factors, besides Ran-GTP.

Interestingly, we demonstrated that HURP molecules photoactivated at the chromosome zone moved poleward, faster than MT flux (Figure 3), while HURP molecules photoactivated near the pole moved towards the equator (Figure 4), indicating bi-directional movement of HURP molecules on the metaphase spindle. This bi-directional movement of HURP is further supported by our stochastic simulation (Supplementary Text and Supplementary Figures S6, S7). The poleward movement of HURP suggests an interaction with a minus-end directed motor protein. Similar photoactivation experiments in mammalian cells have shown that TPX2 is also transported towards the spindle poles faster than the MT flux in a Dynein-dependent manner, and this transport is mediated via Eg5 (Ma et al., 2010). On the other hand, HURP movement in the opposite direction of the MT flux indicates an interaction with a plus-end directed motor protein. Therefore, we propose that HURP can participate in at least two distinct complexes during metaphase.

We found that in mitotic mammalian cells, HURP forms a complex with TPX2, Aurora A, Dynein, Kif5B and, Eg5 (Figure 5 and Supplementary Figure S3A). Aurora A assumes its hyperactive form close to the spindle poles when it is both auto-phosphorylated and bound to TPX2 (Bayliss et al., 2003; Bayliss et al., 2017). We and other have shown that the expression of a kinase-dead Aurora A mutant disrupts a high molecular weight complex of HURP, suggesting that Aurora A-dependent phosphorylation regulates the interaction with its binding partners (Yu et al., 2005; Koffa et al., 2006).

Aurora A-dependent phosphorylation of HURP regulates its MT binding activity (Wong et al., 2008), and its proper localization (Kesisova et al., 2013; Wu et al., 2013). Here, we showed that phosphorylation of HURP, particularly at the Ser627 residue, is essential for proper localization in the vicinity of chromosomes (Figure 6B). The dephospho-mimetic HURP S627A mutant exhibited stable binding to spindle MTs and abolished interaction with Importin *ß* and Kif5B (Figures 6C–F). The loss of Importin *ß* binding for the HURP S627 mutant suggests that both MTBDs of the dephospho-mimetic mutant can be used for spindle MT binding. This is consistent with the findings of Tsuchiya et al., where HURP re-localizes and strongly binds on the MTs near the spindle poles in the absence of Importin *ß*. Moreover, the loss of interaction between HURP S627A mutant and Kif5B may prevent HURP movement towards the chromosomes.

**FIGURE 7:**
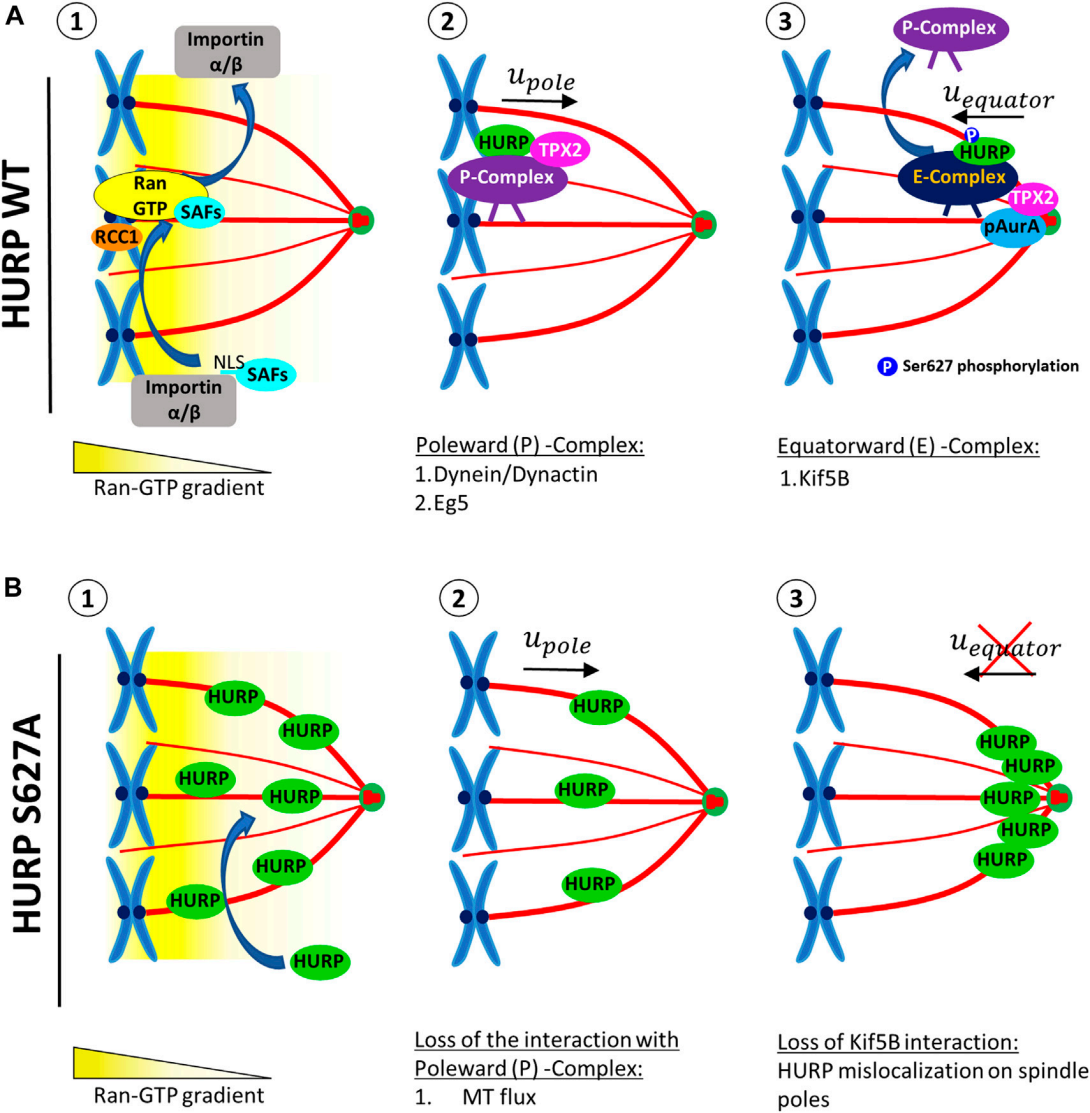
A proposed model for the localization of HURP in the vicinity of chromosomes on the metaphase spindle, involving a two-step process. **(A)** In the presence of high Ran-GTP concentration (1), SAFs including HURP are released from the inhibitory binding of Importins α/β near the chromosomes. HURP, along with other MAPs and motors, bind to MTs and forms the Poleward Complex (P-Complex). The P-Complex includes HURP, TPX2, Dynein/Dynactin, and Eg5. It is then transported towards the spindle pole in a Dynein/Dynactin-dependent manner (2). As the P-Complex reaches the intermediate zone, it interacts with the active Aurora A kinase (pAurA). TPX2 promotes hyperactivation of Aurora A, leading to the phosphorylation of HURP, at least at the Ser627 residue. This phosphorylation event disrupts the Poleward Complex and a new HURP complex is formed (Equatorward (E)-Complex), now containing at least a kinesin, such as Kif5B, which moves towards the equator (3). **(B)** The HURP S627A mutant molecules can bind to MTs independently of the Ran-GTP gradient due to the loss of inhibitory binding of Importin *β* to HURP (1). These mutant molecules bound to the spindle move towards the poles, possibly due to MT flux (2). However, since the mutant HURP molecules are unable to be phosphorylated at Ser627 by Aurora A and cannot interact with Kif5B, they cannot form an E-Complex and instead accumulate at the spindle poles (3).

Based on our findings, we propose a model for the temporal and spatial regulation of HURP (Figure 7). Close to chromatin, Ran-GTP releases SAFs, such as HURP, TPX2 and, Eg5, from Importin α/β inhibition (Figures 7A-1). Released SAFs can then bind to MTs and form a Poleward (P) Complex, which is initially transported towards the spindle poles in a Dynein-dependent manner (Figures 7A-2). As the complex approaches the spindle pole, TPX2-activated pAurora A phosphorylates HURP, at least at the Ser627 residue. Upon phosphorylation, the Poleward (P) complex is disrupted and HURP forms a new complex [Equatorward (E)-Complex], now moving towards the equator in a kinesin-dependent manner (Figures 7A-3). Eventually, Ser627-phosphorylated HURP is dynamically maintained at kt-fibers, creating a polarized localization pattern in the vicinity of chromosomes.

HURP movement towards the MT plus-end could be mediated via the kinesin Kif5B, which we identified as a new interacting partner of HURP (Figures 5C,D). We observed that Kif5B exhibits a similar localization pattern to HURP, in the vicinity of chromosomes (Supplementary Figure S3B). Silencing of Kif5B results in lagging chromosomes, defects in chromosome segregation, spindle organization (Kidane et al., 2013) and disruption of cytokinesis in chondrocytes (Gan et al., 2019). However, additional experiments are required in order to elucidate whether Kif5B is responsible for the equatorward movement of HURP.

On the other hand, due to the loss of interaction with Importin *β*, the dephospho-mimetic HURP S627A mutant can be loaded on spindle MTs independently of the Ran-GTP (Figures 7B-1). These mutant molecules move with MT flux and accumulate towards the spindle pole (Figures 7B-2). Since the mutant HURP molecules cannot interact with Kif5B, they are actively maintained there, due to their strong binding to MTs (Figures 7B-3)

Spindle formation requires both centrosome and chromosome-dependent MT nucleation pathways (Karsenti and Vernos, 2001). TPX2, which is transported poleward in a Dynein-dependent manner (Ma et al., 2010), acts as a scaffold for several proteins including Xklp2 (*Xenopus* kinesin-like protein 2) (Wittmann et al., 1998), Aurora A (Kufer et al., 2002), Eg5 (Ma et al., 2011b), Kif15 (Tanenbaum et al., 2009) and SAF-A (Ma et al., 2011a), and is required for MT nucleation (Schatz et al., 2003). Depletion of TPX2 prevents spindle formation (Gruss et al., 2002). Recent studies have proposed a mechanism in which TPX2 undergoes phase separation-mediated scaffolding of other proteins, including tubulin, for efficient MT nucleation. TPX2 accumulating at the centrosome as mitosis progresses becomes less dynamic than TPX2 near chromosomes, indicating spatiotemporal changes in TPX2 dynamics (King and Petry, 2020).

Inhibition of HURP leads to decreased MT density on the mitotic spindle but does not prevent spindle formation (Koffa et al., 2006). Therefore, HURP and TPX2 need to coordinate in space and time to form a proper mitotic spindle. We propose that HURP and TPX2 interact only transiently within the Poleward complex. When reaching the spindle pole, TPX2 activates Aurora A kinase, leading to the phosphorylation of HURP, particularly at the Ser627 residue. This phosphorylation event triggers a switch in binding partners for HURP. HURP could now interact with a kinesin that transports the protein towards the equator, where it eventually bundles and stabilizes kt-fibers.

It has been recently shown that HURP accumulates on kt-fibers in a manner that is inversely proportional to their length (Dudka et al., 2019). In our experiments, we did not observe asymmetric accumulation of HURP as we only examined centrosome symmetric bipolar spindles. However, the asymmetric accumulation of HURP on the shorter kt-fibers of the acentrosomal half-spindles could also be explained by the reduced MT poleward flux observed in these shorter kt-fibers, thereby increasing the speed at which HURP accumulates at the vicinity of chromosomes, according to our two-step model. Additionally, the formation of Poleward (P) and Equatorward (E) complexes of HURP could supplement the model proposed by Dudka et al., 2019 (Dudka et al., 2019) regarding how centrosomes regulate kt-fiber plus ends through polarized accumulation of HURP.

Moreover, the local cycling model proposed by (Tsuchiya et al., 2021) for explaining HURP’s polarized localization, can be enriched by the two-step model proposed here to include the phosphorylation of HURP at the Ser627 residue by Aurora A before it reaches its final localization on kt-fibers. Additionally, our model also explains the targeting of other Ran-regulated proteins, such as TPX2, at sites spatially separated from chromosomes, thereby linking HURP’s localization near the chromosomes with additional parallel pathways that activate SAFs located distantly from the chromosomes.

In conclusion, we propose that HURP is spatially and temporally regulated by multiple mechanisms to accurately localize to kt-fibers: the Ran-GTP gradient, critical phosphorylation at the Ser627 residue, and protein-protein interactions. Mis-localization of HURP across the metaphasic spindle leads to the formation of a defective spindle that cannot faithfully segregate the chromosomes into the two daughter cells. This, in turn, can result in increased chromosomal instability, closely associated with carcinogenesis and cell malignancy. Gaining insight into how MAPs and motors interact in a coordinated fashion will help us understand how Ran regulates a dynamic mitotic spindle in time and space for proper chromosome segregation. Furthermore, this knowledge will provide additional pharmaceutical targets to intervene and repair potential mitotic defects that may trigger carcinogenesis.

## Summary

Interactions among spindle assembly factors play a crucial role for a faultless mitotic spindle formation. Here, by exploring the spatiotemporal dynamics of HURP, a Ran-GTP regulated microtubule associated protein, we show that HURP bound on spindle microtubules can move in both directions: poleward and equatorward. We propose that the precise chromosome-proximal localization of HURP on kinetochore fibers is the result of a multi-step process that involves interactions with different binding partners. Furthermore, our results highlight the significance of phosphorylation at the Ser627 residue in regulating this process.

## Data Availability

The original contributions presented in the study are included in the article/Supplementary Material, further inquiries can be directed to the corresponding author.
