# Supplementary figures and images for: HURP localization in metaphase is the result of a multi-step process requiring its phosphorylation at Ser627 residue
Source: Front Cell Dev Biol. 2023 Jul 5;11:981425. doi: 10.3389/fcell.2023.981425 (PMC10361663; doi:10.3389/fcell.2023.981425)

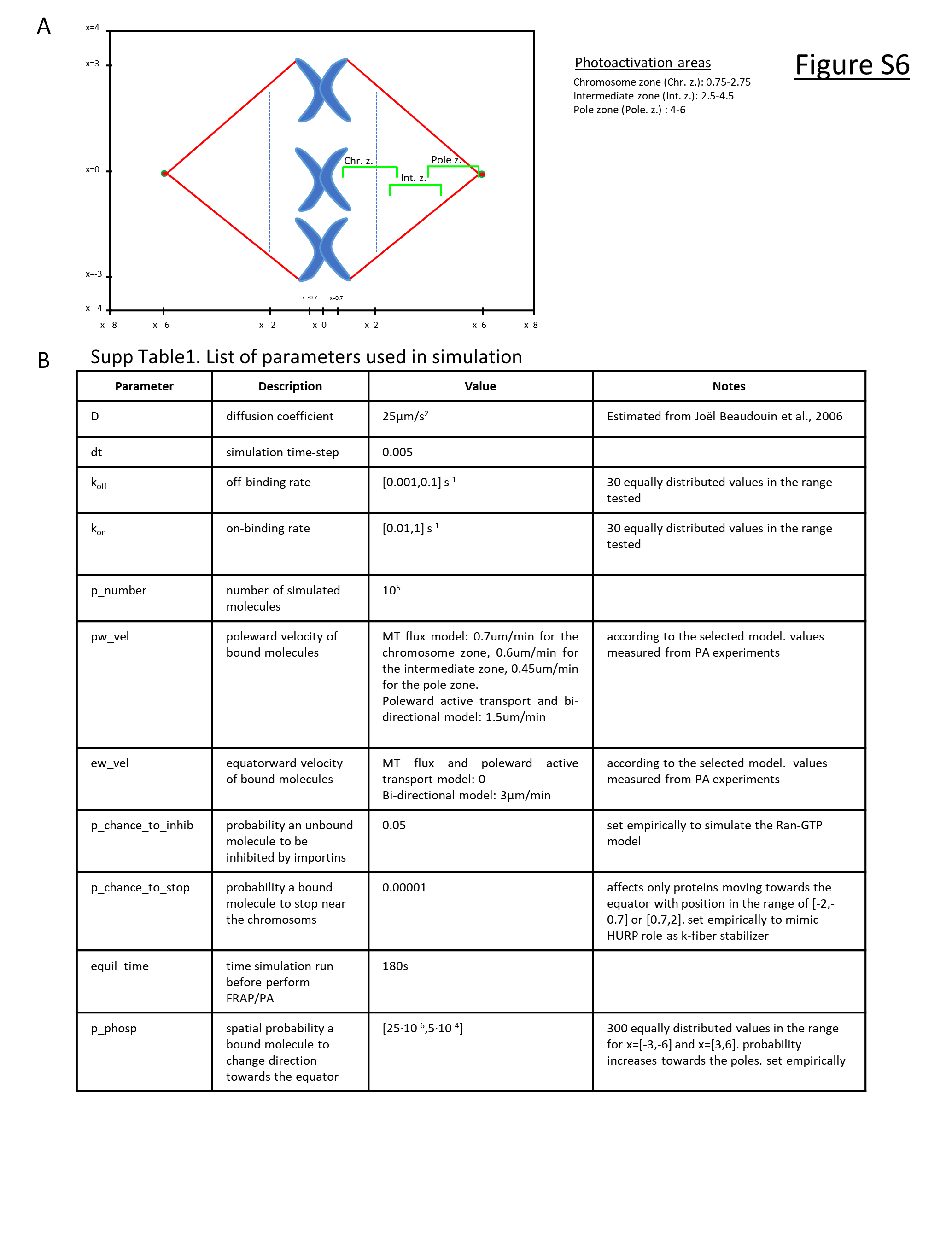

Supplement: Supplementary file 1 [file Image6.tif]

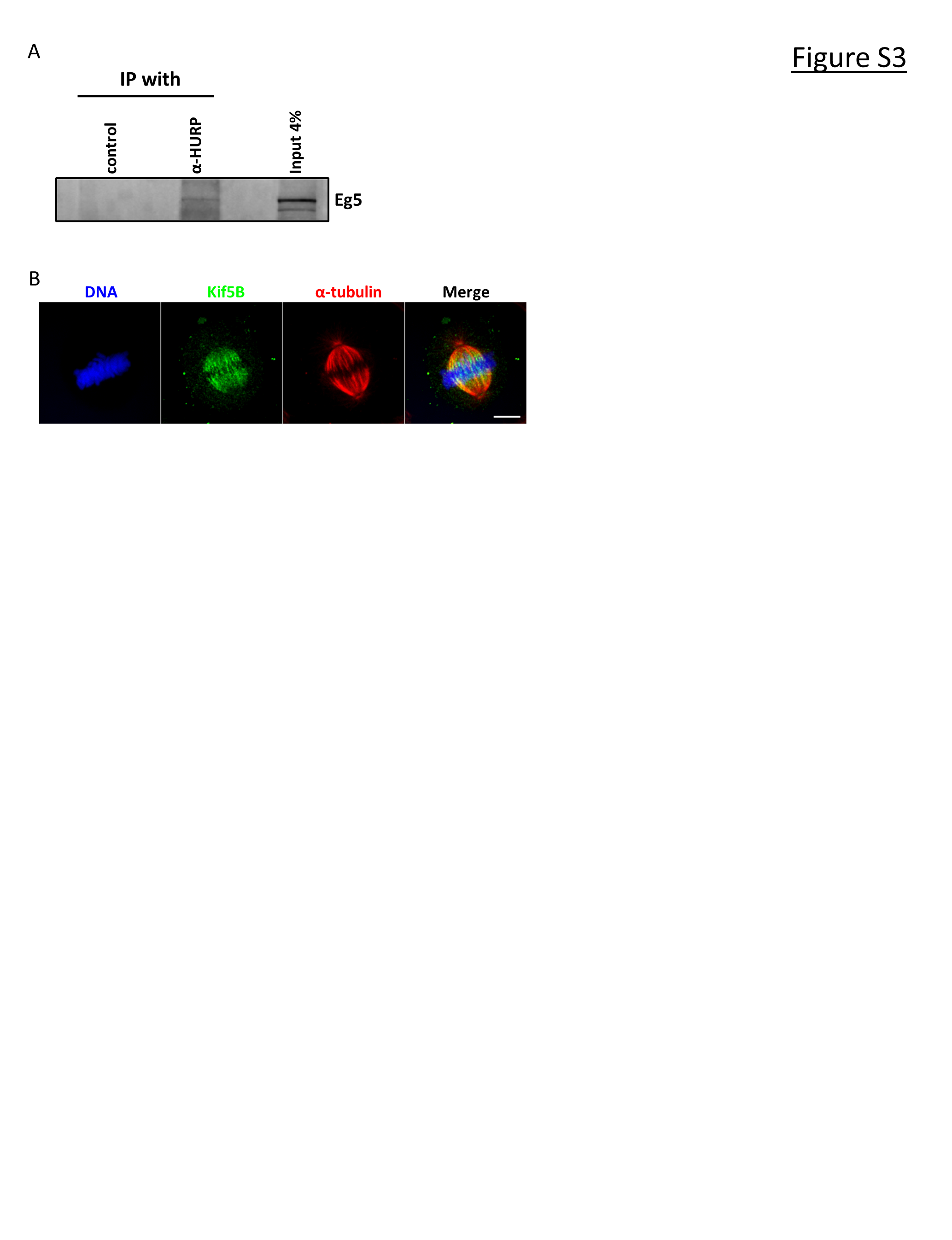

Supplement: Supplementary file 3 [file Image3.tif]

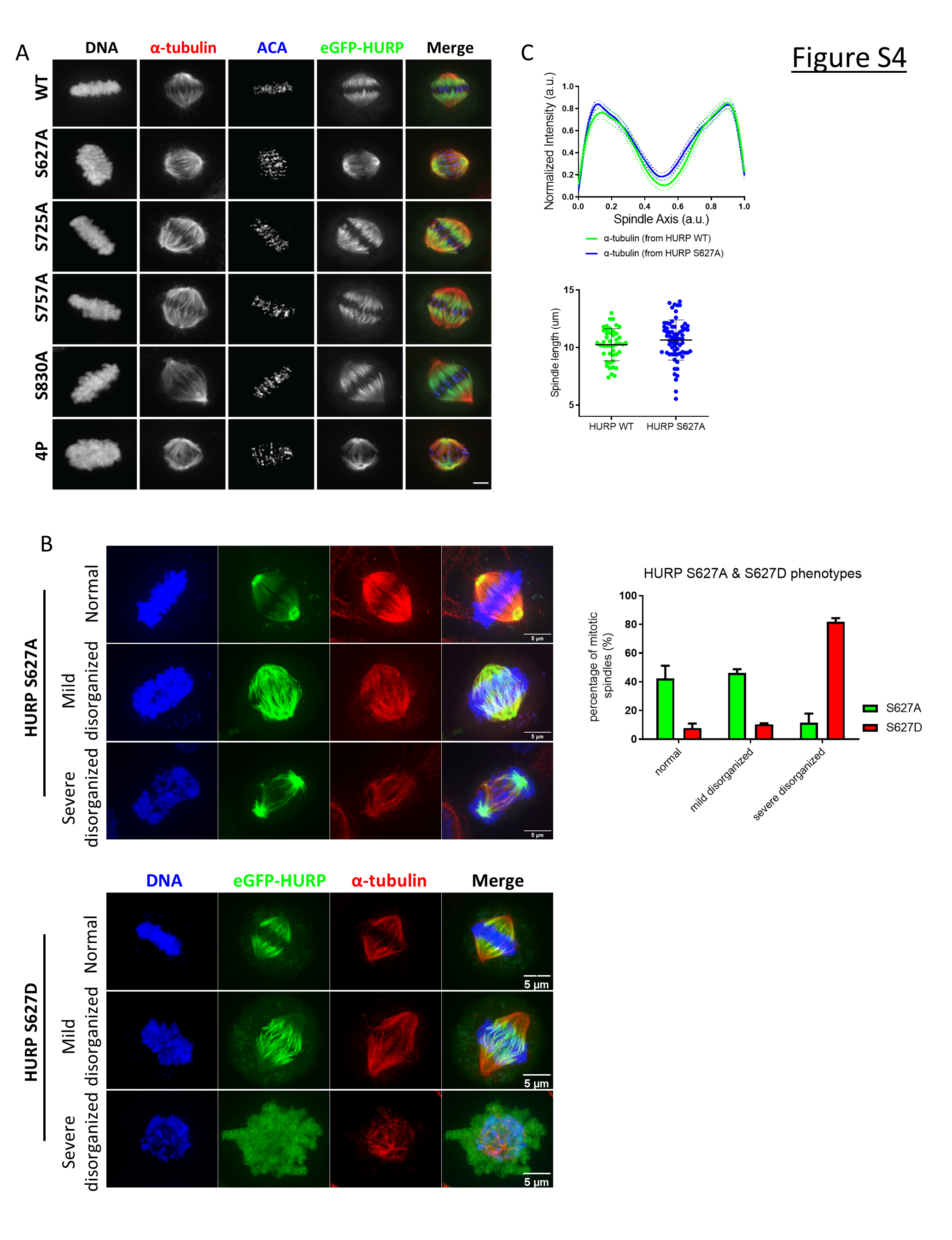

Supplement: Supplementary file 4 [file Image4.tif]

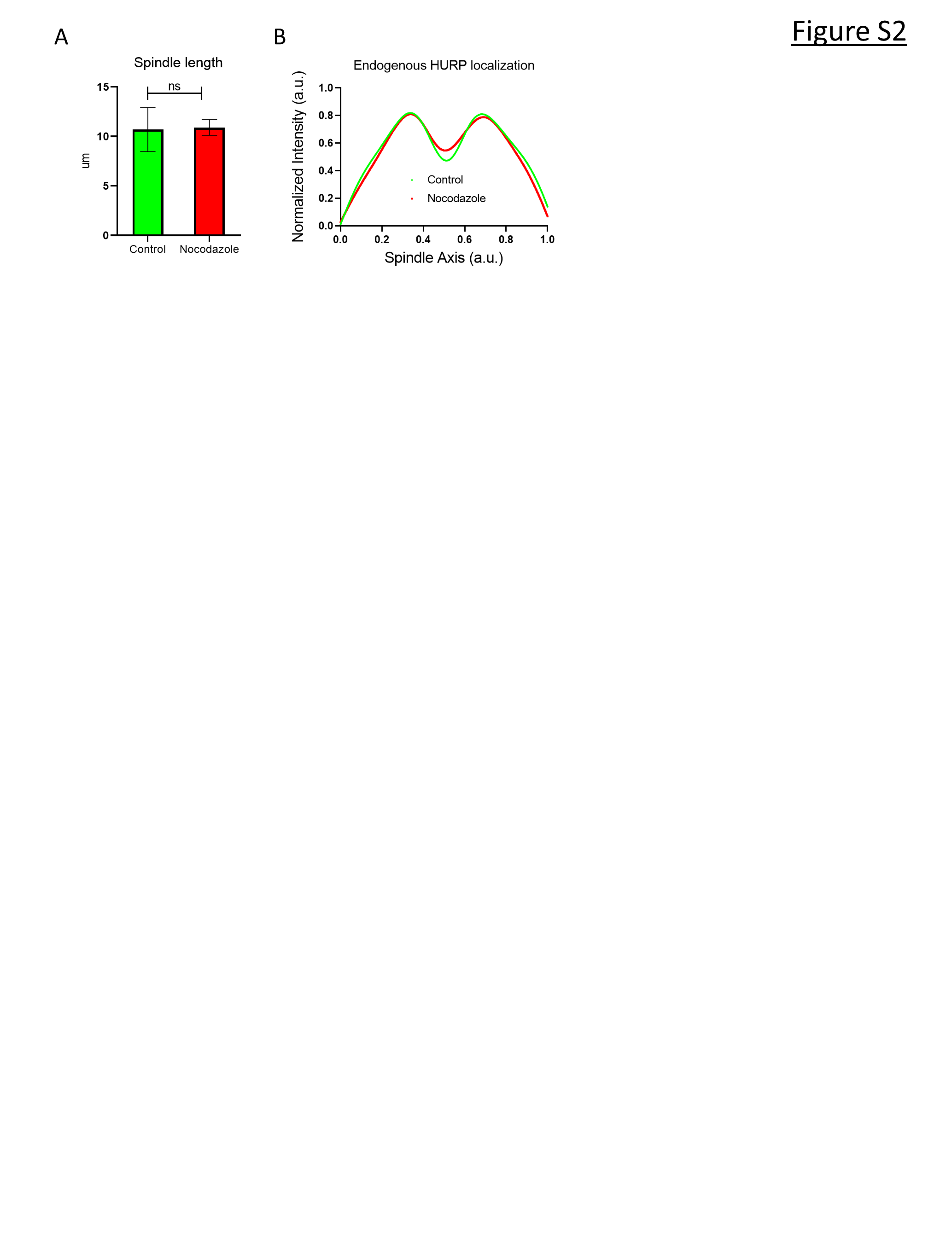

Supplement: Supplementary file 5 [file Image2.tif]

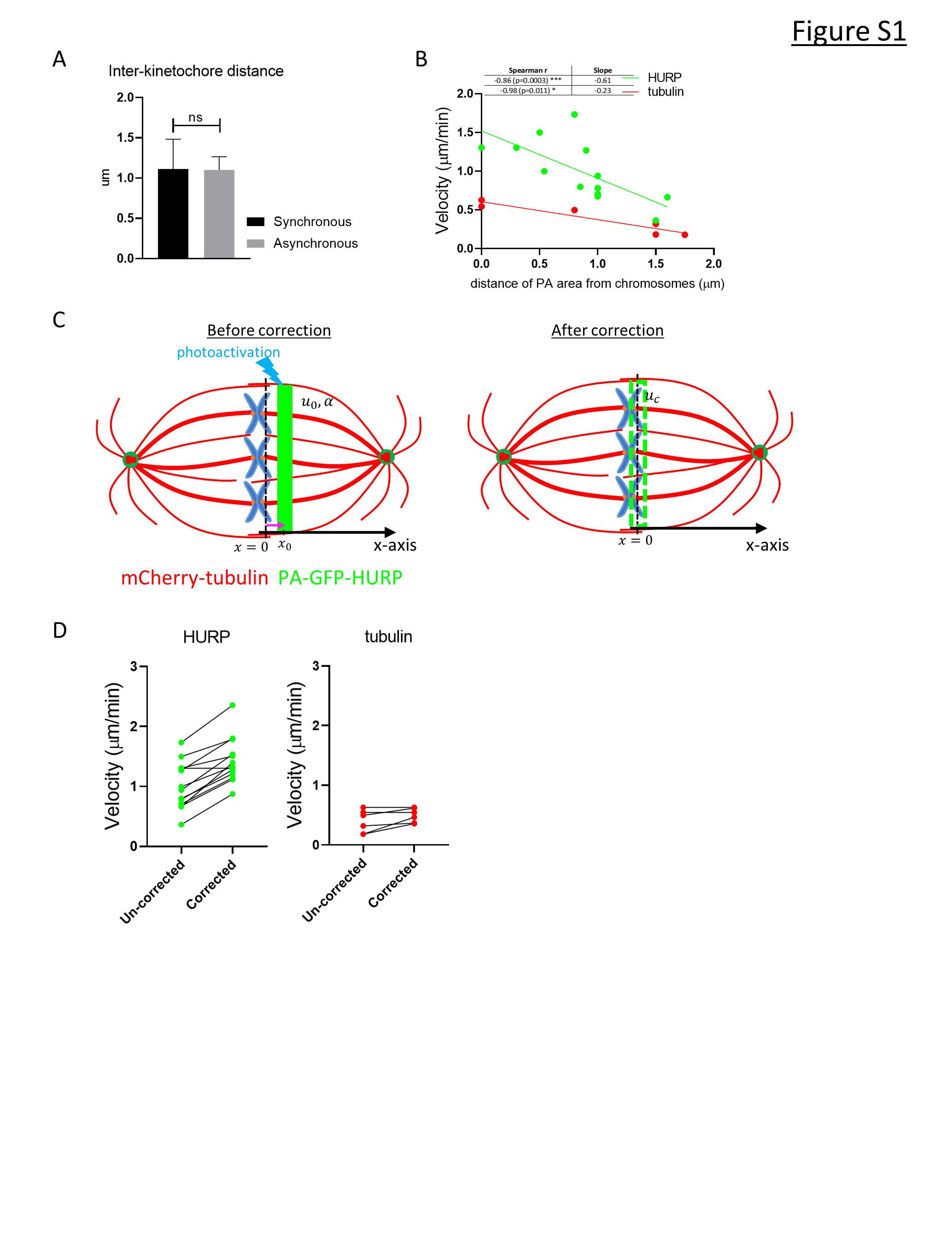

Supplement: Supplementary file 6 [file Image1.tif]

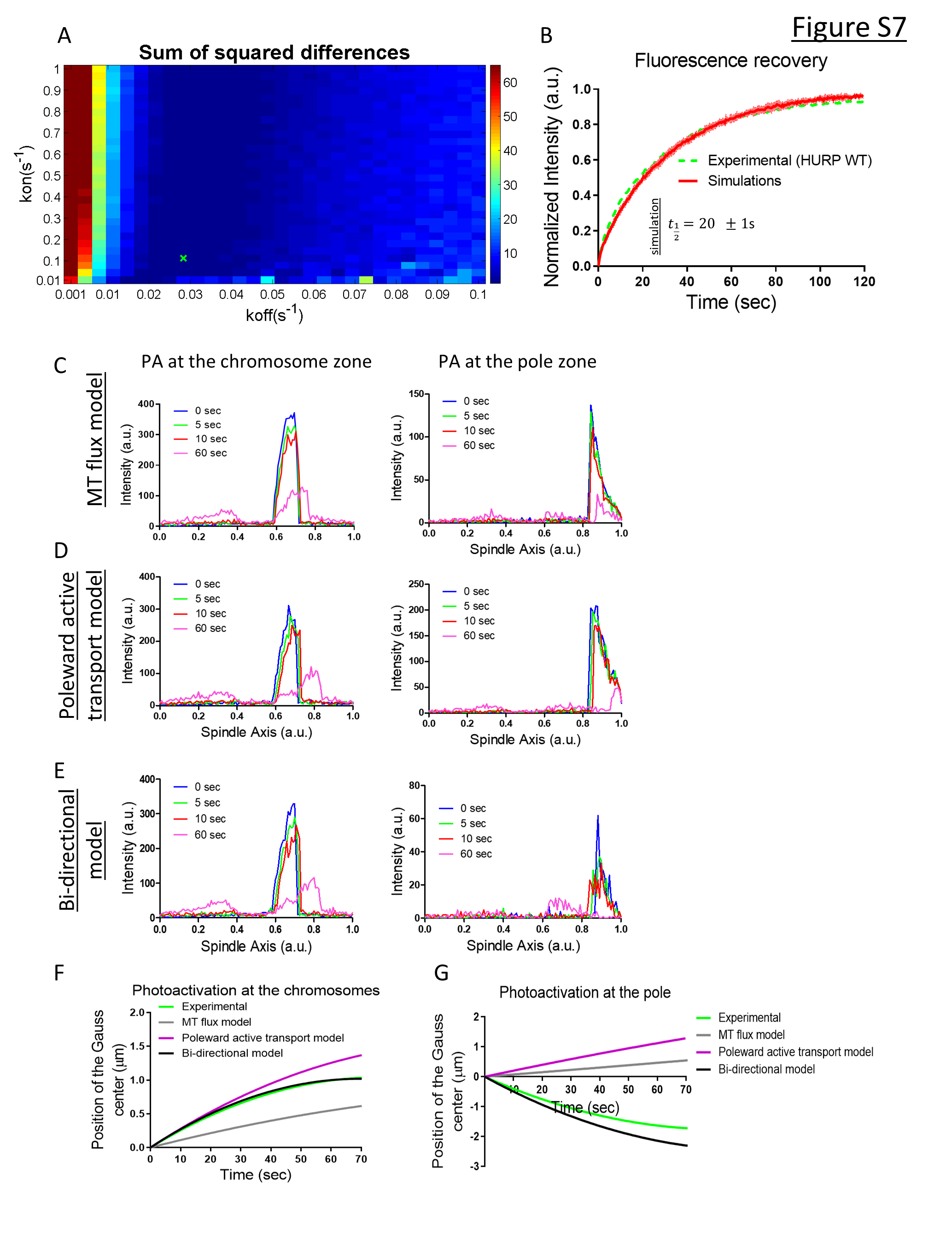

Supplement: Supplementary file 7 [file Image7.tif]

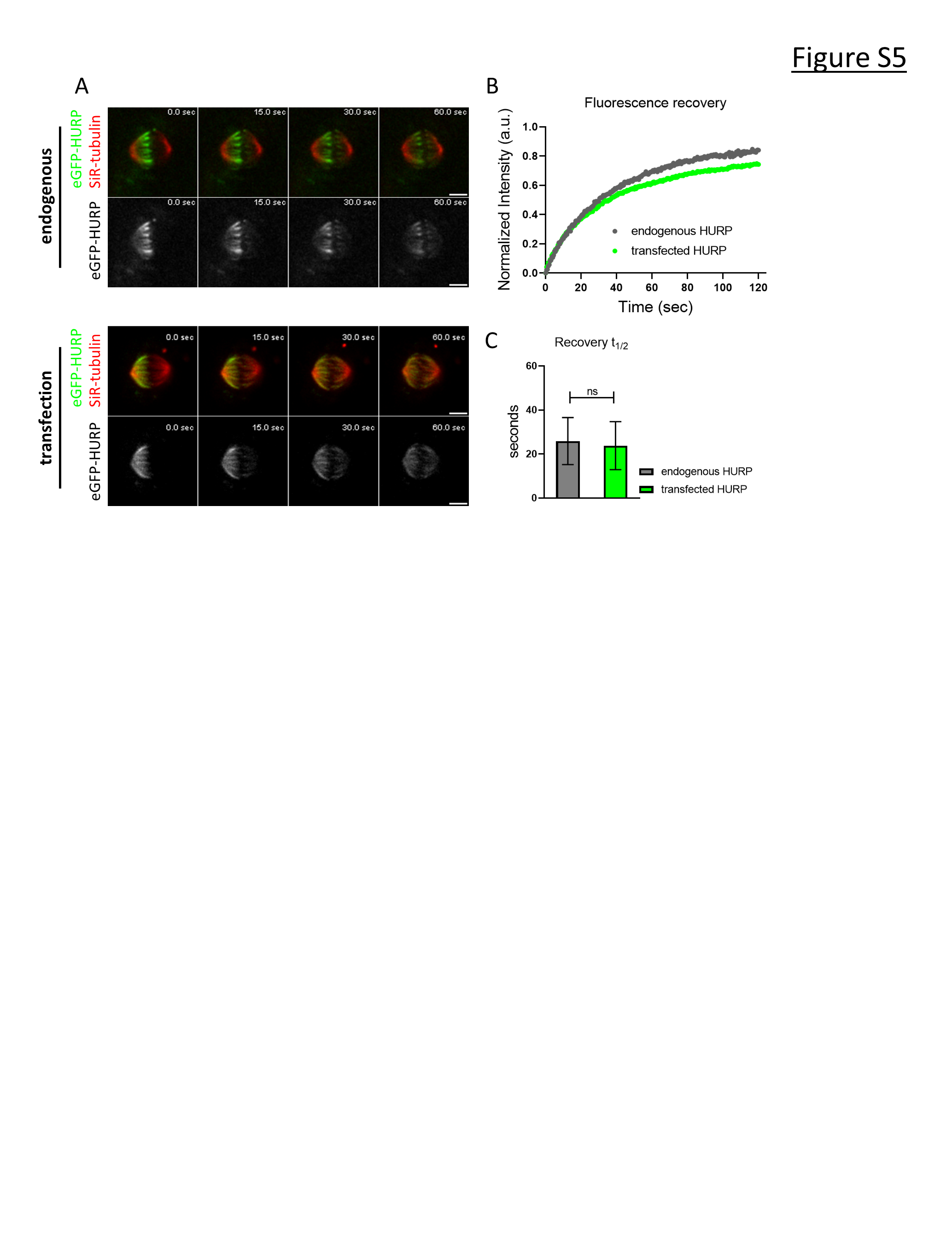

Supplement: Supplementary file 8 [file Image5.tif]
