# Supplementary material for: HURP localization in metaphase is the result of a multi-step process requiring its phosphorylation at Ser627 residue
Source: Front Cell Dev Biol. 2023 Jul 5;11:981425. doi: 10.3389/fcell.2023.981425 (PMC10361663; doi:10.3389/fcell.2023.981425)
Supplement: Supplementary file 2 [file Presentation1.pdf]

Title: HURP localization in metaphase is the result of a multi-step process requiring its phosphorylation at Ser627 residue

Authors: Stylianos Didaskalou[1,†], Christos Efstathiou[1,2,†], Sotirios Galtsidis[2], Ilona Kesisova[1,2], Aliaksandr Halavatyi[3], Tountzai Elmali[1], Avgi Tsolou[1], Andreas Girod[2], Maria Koffa[1*]

[1]Department of Molecular Biology and Genetics, Democritus University of Thrace, Alexandroupolis, Greece

[2]Department of Life Sciences and Medicine, University of Luxembourg, Esch-sur-Alzette, Luxembourg

[3]Advanced Light Microscopy Facility, European Molecular Biology Laboratory, Heidelberg, Germany

ORCID: Stylianos Didaskalou: 0000-0001-5932-9626, Christos Efstathiou: 0000-0001-8964-0423, Sotiris Galtsidis: 0000-0001-7001-9944, Ilona Kesisova: -, Aliaksandr Halavatyi: 0000-0002-9002-457X, Tountzai Elmali: 0000-0003-1836-6273, Avgi Tsolou: 0000-0002-0139-019X, Andreas Girod: -, Maria Koffa: 0000-0003-2255-3426

†These authors contributed equally to this work and share first authorship

***Correspondence: Maria Koffa, Department of Molecular Biology and Genetics, Democritus University of Thrace, Building 10, Dragana, 68100, Alexandroupolis, Greece. email: mkoffa@mbg.duth.gr, Tel: 00302551030661**

Simulations of the bi-directional movement of HURP on metaphase spindle

We implemented a 2D stochastic simulation in which FRAP or photoactivation experiments can be performed, using three different kinetic models: a) HURP molecules bind close to the chromosomes due to the Ran-GTP gradient, on fluxing MTs (MT flux model), b) MT-bound HURP molecules are actively transported poleward (active transport model) and c) MT-bound HURP molecules are initially transported poleward, but before they reach the spindle pole they switch direction, and they are eventually transported towards the equator (bi-directional model). The switch in the movement's direction is a stochastic process, with increasing probabilities as molecules approach the pole zone.

For all models, 10^5 individual molecules were simulated, stochastically switching between unbound and MT-bound state in the continuous space (cell size 16x8μm, spindle size 12x6μm) (see Supplementary Text Methods). A schematic geometry of the spindle as well as a table with constants used in the simulation is shown in Figure S6.

To mimic the RanGTP gradient, HURP molecules are considered released by importin β only at the chromosomes-proximity (chromosome zone, see Figure S6). In each simulation time step a released molecule can either diffuse (if in the unbound state), bind to MTs, or become inhibited again by importin β. Once bound to MTs, the molecules can either unbind or move on half spindle along fluxing MTs, according to the selected kinetic model. For all simulations molecules were initially set to be inhibited by importin β; thus, the system ran for 180 seconds to reach a steady state before performing FRAP or photoactivation experiments.

To find the binding and unbinding reactions rates of HURP molecules on spindle MTs, we fitted the MT flux model with the acquired FRAP data by testing different k_{on} and k_{off} values. Data from simulated FRAP were normalized and the sum of squared differences between experimental and simulated data were calculated (Figure S7A). Figure S7A shows that HURP fluorescence recovery is mainly affected by the off-rate rather than the on-rate, in the selected range of values. The pair with the lower sum of squared residuals ($k_{on} = 0.11s^{-1}$, $k_{off} = 0.028s^{-1}$) produced recovery curves with $t_{1/2}$ very close to the experimental data (Figure S7B), and was therefore selected for further use. Using these values as k_{on} and k_{off} rate for MT binding and unbinding of HURP molecules, photoactivation simulations were performed. For each of the three different kinetic models, photoactivation at both the chromosome and the pole zone was simulated (Figure S7C-G) and the results were compared to the experimental photoactivation data (Figures 3 and 4).

Testing the first kinetic model, where HURP molecules bind close to the chromosomes on fluxing MTs, simulated molecules photoactivated at the chromosome zone move poleward slower than experimentally observed (Figure S7C; left, Figure S7F), whereas simulated molecules photoactivated near the pole zone continue to move poleward the (Figure S7C; right), and not equatorward, as experimentally observed (Figure S7G).

Examining the second kinetic model, where MT-bound HURP molecules move poleward by active transport (with $u_{poleward} = 1.53$ as measured experimentally), the simulated molecules photoactivated at the chromosome zone move similarly to the experimental (Figure S7D; left). However, the model fails to predict the change in the slope of the fitted curve (Figure S7F). Simulated molecules photoactivated at the pole zone (Figure S7D; right) continue to move poleward, in contrast to the experimental data (Figure S7G).

Simulations performed according to the third kinetic model, where MT-bound HURP molecules move bi-directionally, showed that the simulated molecules photoactivated at the chromosome zone move initially poleward, as the experimental data (Figure S7E; left), predicting also the change in the slope of

the fitted curve (Figure S7F). Moreover, simulated molecules photoactivated at the pole zone move towards the equator, as demonstrated experimentally (Figure S7G). This simulation model failed to predict the accurate change in the slope after the first 40s.

Overall, our simulations show that neither the MT flux alone nor the active poleward transport models can predict the experimental data. Conversely, the assumption of bi-directional movement of HURP can much better reproduce the experiments *in silico*.

Methods

Stochastic simulation

For the simulations performed the algorithm was written in MATLAB. Cells margins were set as a rectangle with sides of 16x8μm and fluxes were set to zero across the cell's membrane. A schematic geometry of the spindle as well as a table with constants used in simulation are available in Figure S6.

The $n = 10^5$ individual particles were simulated in the continuous space (cell size 16x8μm, spindle size 12x6μm) with discrete time steps of dt = 0.005 seconds. Molecules could stochastically switch between unbound and MT bound state, with probabilities given by the equations (Roding et al., 2019):

$$p_{u \to b} = dt * k_{on} \qquad (Equation\ 1.1)$$

$$p_{b \to u} = dt * k_{off} \qquad (Equation\ 1.2)$$

where k_{on} and k_{off} are the on- and off-rates ($in\ s^{-1}$), accordingly. Infinite binding sites were assumed. The initial position of particles was uniformly set inside the cell margins. At each time step, if a molecule is in the unbound state, it is displaced by $\sqrt{2Ddt}$ in the randomly selected direction, where $D = 25\frac{\mu m^2}{s}$ is the diffusion coefficient.

When simulating FRAP experiments, the readout was collected at time intervals of Δt_{FRAP}=0.5s, matching the time resolution of real experiments. Five pre-photobleaching time frames were simulated. Then spindle bound molecules and molecules in the area of the half spindle were marked as photobleached and their recovery was simulated for 240-time frames. The number of the recovered HURP molecules over the whole spindle as well as in the photobleached area were recorded at each time point. To obtain recovery curves, data were normalized as in experiments.

In simulations of PA experiments, 5 pre-photoactivation and 22 post-photoactivation frames were acquired with time interval of Δt_{PA}= 5s. Photoactivation was simulated in a 2μm wide area at the

chromosome zone, the pole zone and the intermediate zone. A 2μm wide pole–to-pole line was used to measure the number of photoactivated molecules over the spindle and to quantify the movement rates applying the same analysis used for the experimental data.

In each simulation time step a free molecule can diffuse, if it is in the unbound state, get bound if it is over the mitotic spindle, or get inhibited again by importin β. A bound molecule can unbind or move across the mitotic spindle according to the selected kinetic model. In the "MT flux only" kinetic model, bound molecules were set to move towards the spindle poles with same velocity as MT flux (u_{flux}) as measured from PA experiments (Figure 3E-tubulin), mimicking a passive transport due to MT flux. Half of the mitotic spindle were compartmentalized in three regions, as in PA experiments, and for each compartment different MT flux velocity was set, to account for the decreasing MT flux, as approaching the poles. In the active poleward transport, bound molecules were set to move towards the spindle poles faster than MT flux, mimicking the dynein dependent active transport of the protein. The poleward velocity was set equal to what measured in PA experiments ($u_{poleward}$, Figure 3E-HURP). In the full model, bound molecules can move either towards the spindle poles or towards the equator. The velocities were set as measured from PA experiments ($u_{poleward}$, and $u_{equatorward}$, accordingly). Transition between poleward to equatorward movement is a stochastic process with higher probabilities as molecules approach poles. In all cases, when a bound molecule reaches the pole, it gets unbound. When a bound molecule moving towards the equator, it may stop if it reaches close to the chromosomes (in area range of [-2,-0.7] for left half spindle or in area range of [0.7,2] for the right half spindle), or get unbound if it reaches the plus end of MTs (-0.7 for left half spindle or 0.7 for right spindle). To take into consideration the possible movement of molecules during photobleaching and photoactivation, the system is simulated for 60dt=0.3 seconds before acquiring the first post FRAP/PA event.

Supplementary figure legends

Figure S1.

A) Inter-kinetochore distance in HeLa Kyoto cells at metaphase. Distances were measured between pair centromeres after ACA staining. (n = 159 pairs for synchronous and n = 79 pairs for asynchronous).
B) Measured initial velocities of PA-GFP-HURP and PA-GFP-tubulin, (u_o), extracted by fitting the center of fluorescence distribution over time, were found to negatively correlate with the

distance of photoactivation (PA) area from the chromosomes. Slope was calculated by fitting a linear model (PA-GFP-HURP, n =13 cells; PA-GFP-tubulin, n = 6 cells).

C) Procedure followed for correcting the initial velocity u_0 to theoretical velocity u_c. The velocity of PA-GFP-HURP (u_0) and acceleration a (change of slope) were extracted by fitting $x - t$ data points with a second order polynomial. The theoretical velocity u_c at the edge of the chromosomes (x=0), was calculated using Equations 1.1 and 1.2 (see Materials and Methods).

D) Velocity-pairs, before and after correction (PA-GFP-HURP, n =13 cells; for PA-GFP-tubulin, n = 6 cells).

Figure S2.

A) Spindle length of HeLa Kyoto cells endogenously expressing eGFP-HURP, in control and Nocodazole (10nM) treated cells, counter stained with SiR-tubulin and arrested in metaphase using MG132 (control, n = 24 cells; Nocodazole, n = 29 cells).

B) Intensity plot profiles along metaphase spindle long axis in HeLa Kyoto cells endogenously expressing eGFP-HURP. Cells were either control or Nocodazole (10nM) treated, counter stained with SiR-tubulin and arrested in metaphase using MG132 (control, n = 20 cells; Nocodazole, n = 20 cells).

Figure S3.

A) Co-immunoprecipitation assay using an antibody against HURP in HeLa Kyoto mitotic cell extracts. Rabbit IgG was used as control. HURP bound complexes were analyzed by Western blot analysis using an antibody against Eg5. Input represents 4% for Eg5.

B) Immunofluorescence analysis of Kif5B in HeLa Kyoto mitotic cells after pre-extraction with digitonin. Immunofluorescence has been performed against Kif5B and α-tubulin, DNA was counterstained with Hoechst. Scale bars denote 5μm.

Figure S4.

A) Representative immunofluorescence images of metaphasic HeLa Kyoto cells transfected with eGFP-HURP WT or an eGFP-HURP mutant, as indicated (S627A, S725A, S757A, S830A). 4P corresponds to a mutant where all 4 residues (S627, S725, S757, S830) are replaced by Alanine. Immunofluorescence has been performed against α-tubulin and centromeres (ACA). DNA is counterstained with DAPI. In the merged image ACA is pseudo-colored blue, α-tubulin red and HURP green. Scale bar denotes 5μm.

B) Representative immunofluorescence images of mitotic HeLa Kyoto cells transfected with either eGFP-HURP S627A (upper panel) or eGFP-HURP S627D (lower panel) mutants. The mitotic phenotypes were categorized, based on MTs organization and chromatin localization in three different phenotypes: normal, mild disorganized and severe disorganized spindles. Scale bars denote 5μm.

Right: Quantification of the different phenotypes on spindle organization observed upon eGFP-HURP S627A or eGFP-HURP S627D mutant transfection in HeLa Kyoto cells. (HURP S627A, n = 165 cells; HURP S627D, n = 123 cells). Normal spindle organization was observed in 42.3%, mild disorganization in 46.2% and severe disorganization in 11.5% of cells expressing eGFP-HURP S627A. In contrast, normal spindle organization was observed in 7.7%, mild disorganization in 10. 5% and severe disorganization in 81.8% of cells expressing eGFP-HURP S627D.

C) Top: Tubulin pole-to-pole intensity plot profiles in metaphasic HeLa Kyoto cells transfected with either eGFP-HURP WT or eGFP-HURP S627A mutant (normal spindle phenotype). Bold-lines indicate the mean values and dashed-lines represent the ± S.D. (HURP WT, n=46 cells; HURP S627A, n=69 cells).

Bottom: Scatter plot showing the mean spindle size of HeLa Kyoto metaphasic cells transfected with either eGFP-HURP WT or eGFP-HURP S627A mutant (normal spindle phenotype). Error bars indicate ± S.D. (HURP WT, n=46 cells; HURP S627A, n=69 cells).

Figure S5.

A) Representative images of FRAP experiments on HeLa Kyoto metaphasic cells expressing eGFP-HURP either at endogenous levels (upper panel) or transiently overexpressed (lower panel). Cells were counterstained with SiR-tubulin. Scale bars denote 5μm.

B) Fluorescence recovery curves of eGFP-HURP. Curves show the mean of n = 24 for endogenous and n = 18 for transiently expressed (see Figure 1B for bleaching geometry).

C) Quantification of FRAP curves by one-phase exponential fitting. Mean values with ± S.D. are shown (endogenous, n = 24 cells; transfected, n = 18; ***p=0.0003).

Figure S6. Schematic representation of spindle and list of parameters used in simulation.

A) Geometry of simulated spindle. Black box represents the cell margins with its dimensions. Red lines represent peripheral MTs that define the spindle area where molecules can bind (x= [-6,6],

y= [-3,3]). Blue dotted lines from [-2,2] define the area where molecules are released from inhibitory binding of Importins, mimicking the RanGTP gradient. In FRAP, all molecules over the spindle in the range of x= [0,6] were marked as photobleached. The three different photoactivated zones are shown in green. The exact coordinates of each zone are shown in the top right.

B) Table with a list of parameters used and their values.

Figure S7. FRAP and Photoactivation simulations

A) Heatmap showing the sum of squared residuals between experimental and simulated fluorescence recovery curves as a function of $k_{on}$and k_{off} values. The first model of MT flux was used to fit the acquired FRAP data, in order to find rates of MT binding and unbinding reactions. 900 pairs of 30 different k_{on} and k_{off} values were tested. For each pair, the FRAP recovery data after photobleaching half spindle was simulated, normalized, and the sum of squared differences between experimental and simulated data were calculated. Sum of squared residuals is encoded with color-coding, as shown by the bar on the right. The pair with the lower sum of squared residual (green "**x**"; $k_{on} = 0.11s^{-1}$, $k_{off} = 0.028s^{-1}$), was chosen for further simulations.

B) Cumulative fluorescence recovery curve after photobleaching obtained either experimentally (green line) or simulated with the selected k_{on} and k_{off} described in A (red line). Simulated readout was collected at time intervals of Δt_{FRAP}=0.5 seconds. 5 pre-photobleaching time frames were simulated, followed by photobleaching the half spindle, and the recovery of the photobleached molecules was simulated for 240-time frames. The number of the recovered HURP molecules over the whole spindle as well as in the photobleached area were recorded at each time frame. To obtain recovery curves, data were normalized as in experiments. n=7 simulations were performed and fitted with a two-phase association curve. Average values of $t_{1/2}$ of the two phases of the simulated curve are shown (mean ± S.D), 95% confidence intervals are shown inside brackets.

C) Simulation outcomes for the "MT flux" model. In simulations of photoactivation experiments, 5 pre-photoactivation and 22 post-photoactivation frames were acquired with time interval of Δt_{PA}= 5 seconds. Photoactivation was simulated in a 2μm wide area at the chromosome zone, the pole zone and the intermediate zone. A 2μm wide pole–to-pole line was used to measure

the number of photoactivated molecules over the spindle and to quantify the movement rates applying the same analysis used for the experimental data.

Plot profiles of photoactivated molecules near the chromosome zone (left) and near the pole zone (right), are shown. 0 seconds correspond to the first-time frame after photoactivation.

D) Simulation outcomes for the “poleward active transport” model. Plot profiles of photoactivated molecules near the chromosome zone (left) and near the pole zone (right) are shown. 0 seconds correspond to the first frame after photoactivation.

E) Simulation outcomes for the “bi-directional” model. Plot profiles of photoactivated molecules near the chromosomes zone (left) and near the pole zone (right) are shown. 0 seconds correspond to the first frame after photoactivation.

F) Position of the Gauss center versus time for molecules photoactivated near the chromosomes. For each kinetic model data points were fitted with a second order polynomial equation to plot the trendline. Experimental data are shown in green.

G) Position of the Gauss center versus time for molecules photoactivated near the poles. For each kinetic model data points were fitted with a second order polynomial equation. Experimental data are shown in green.

References

Roding, M., L. Lacroix, A. Krona, T. Geback, and N. Loren. 2019. A Highly Accurate Pixel-Based FRAP Model Based on Spectral-Domain Numerical Methods. *Biophys J*. 116:1348-1361.
